# Effectiveness of Case Management for 'At Risk' Patients in Primary Care: A Systematic Review and Meta-Analysis

**DOI:** 10.1371/journal.pone.0132340

**Published:** 2015-07-17

**Authors:** Jonathan Stokes, Maria Panagioti, Rahul Alam, Kath Checkland, Sudeh Cheraghi-Sohi, Peter Bower

**Affiliations:** 1 NIHR Greater Manchester Primary Care Patient Safety Translational Research Centre, Manchester Academic Health Science Centre, University of Manchester, Manchester, United Kingdom; 2 NIHR School for Primary Care Research, Centre for Primary Care, Manchester Academic Health Science Centre, University of Manchester, Manchester, United Kingdom; University of Glasgow, UNITED KINGDOM

## Abstract

**Background:**

An ageing population with multimorbidity is putting pressure on health systems. A popular method of managing this pressure is identification of patients in primary care ‘at-risk’ of hospitalisation, and delivering case management to improve outcomes and avoid admissions. However, the effectiveness of this model has not been subjected to rigorous quantitative synthesis.

**Methods and Findings:**

We carried out a systematic review and meta-analysis of the effectiveness of case management for ‘at-risk’ patients in primary care. Six bibliographic databases were searched using terms for ‘case management’, ‘primary care’, and a methodology filter (Cochrane EPOC group). Effectiveness compared to usual care was measured across a number of relevant outcomes: **Health** – *self-assessed health status*, *mortality*; **Cost** – *total cost of care*, healthcare *utilisation* (*primary and non-specialist care* and *secondary care* separately), and; **Satisfaction** – *patient satisfaction*. We conducted secondary subgroup analyses to assess whether effectiveness was moderated by the particular model of case management, context, and study design. A total of 15,327 titles and abstracts were screened, 36 unique studies were included. Meta-analyses showed no significant differences in *total cost*, *mortality*, *utilisation of primary* or *secondary care*. A very small significant effect favouring case management was found for *self-reported health status* in the short-term (0.07, 95% CI 0.00 to 0.14). A small significant effect favouring case management was found for *patient satisfaction* in the short- (0.26, 0.16 to 0.36) and long-term (0.35, 0.04 to 0.66). Secondary subgroup analyses suggested the effectiveness of case management may be increased when delivered by a multidisciplinary team, when a social worker was involved, and when delivered in a setting rated as low in initial ‘strength’ of primary care.

**Conclusions:**

This was the first meta-analytic review which examined the effects of case management on a wide range of outcomes and considered also the effects of key moderators. Current results do not support case management as an effective model, especially concerning reduction of secondary care use or total costs. We consider reasons for lack of effect and highlight key research questions for the future.

**Review Protocol:**

The review protocol is available as part of the PROSPERO database (registration number: CRD42014010824).

## Introduction

Many health care systems currently face significant pressures resulting from both increasing numbers of older patients with multiple long-term conditions (multimorbidity), and pressure to reduce health care budgets or provide more efficient use of current resources [1].

To relieve these pressures, many policy makers and health system planners advocate ‘integrated care’ [1, 2].

**Fig 1:**
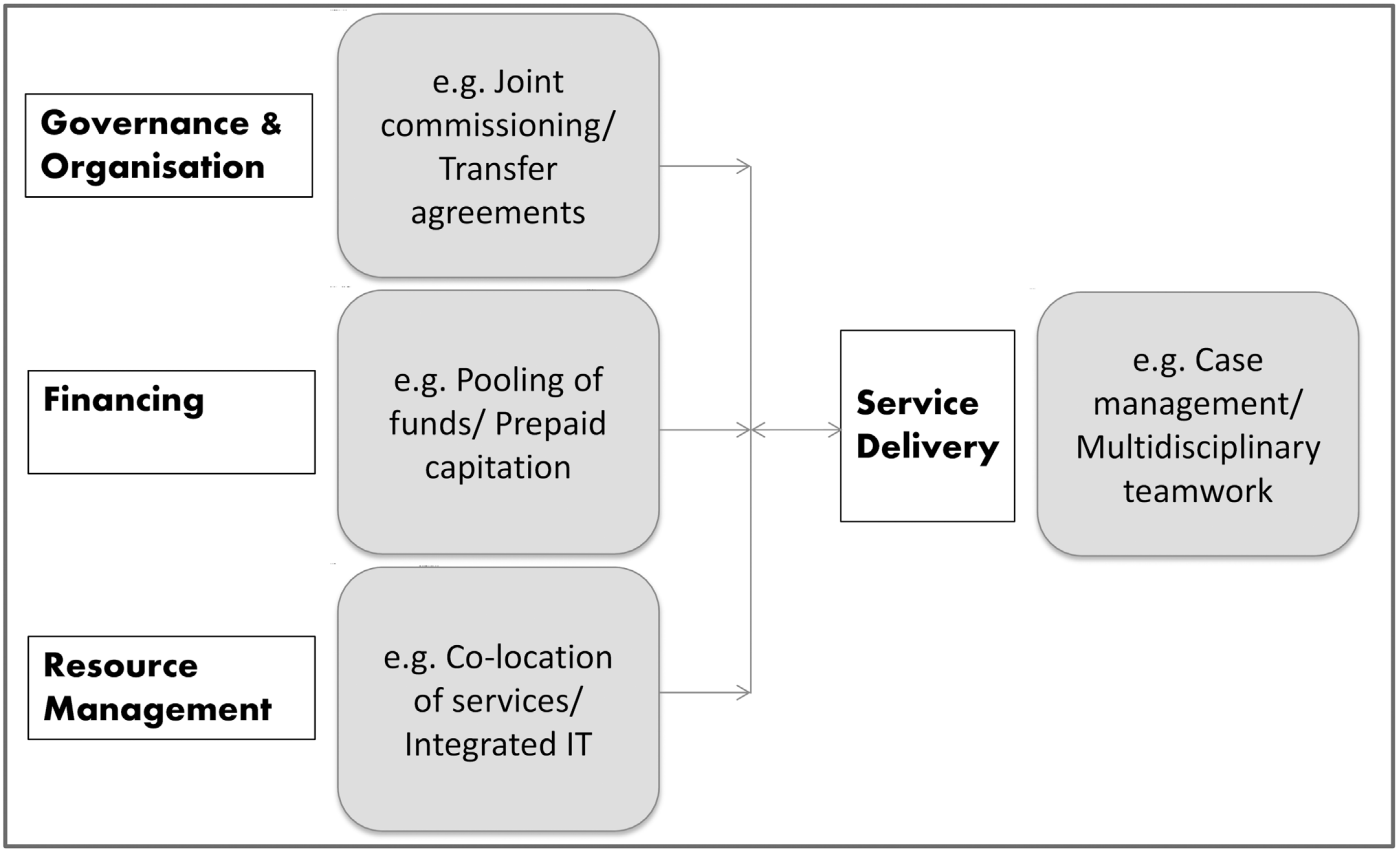
Examples of popular methods to ‘integrate’ care [3] within the health system [5].

Integrated care is a complex concept. Broadly, it is designed to “create connectivity, alignment and collaboration” [3]. A number of different methods can be used to achieve these inter-connections, and they can occur at multiple ‘levels’ of the health system (e.g. financing, resource management, service delivery—see Fig 1). Outcomes of effective integration of care are presumed to be better patient experience and outcomes, as well as greater efficiency [4] (i.e. patient satisfaction; health; and cost-effectiveness), therefore potentially addressing two of the major system pressures simultaneously.

A popular model of ‘integrated care’ at the service delivery level is ‘case management’ in primary care [6, 7]. Case management has been defined as:

“a collaborative process of assessment, planning, facilitation, care coordination, evaluation, and advocacy for options and services to meet an individual’s and family’s comprehensive health needs through communication and available resources to promote quality, cost-effective outcomes” [8].

Variations exist in the delivery of case management. However, there are common components [6]:

- *case-finding* (identifying those ‘at risk’ who require case management, usually through prediction of high costs in the future [9, 10])
- *assessment* of the needs of the individual patient, and *care planning* (individualised care plan bringing together details of patient’s personal circumstances with health and social care needs, and aiming to match these needs with service provision)
- *care co-ordination* (navigational role of case manager involving continual communication with patients, carers, professionals and services e.g. medication management, self-care support, care advocacy and negotiation; with regular review, monitoring and adaptation of the care plan)

Case finding of ‘at-risk’ individuals can be done in three ways [11]:

1. clinical judgement (expert opinion),
2. threshold modelling (defining a set of rules e.g. number of previous hospital admissions, which alert the practitioner that the patient is at risk), or
3. using a predictive risk tool (where an algorithm is used to attempt to *predict* those patients who are at risk of a defined event)

In theory, the case management process may increase efficiency by reducing unnecessary contacts with the health system, including fragmented routine contacts, as well as emergency contacts caused by potentially preventable exacerbations. The goal is to better co-ordinate care, offering individually-tailored contacts and care planning.

Primary care is a suitable context for integrated care due to its place at the heart of the health system [12]. It is argued that increasing care in the community setting will facilitate cost savings compared to expensive hospital overheads [13]. In many health systems, primary care acts as a ‘gatekeeper’ to the rest of the system [14] and primary care practitioners should be particularly suited to managing and co-ordinating care for multiple health problems, compared to specialist physicians [15].

The potential benefits of case management have led to adoption in practice in many countries [6]. For example, in the United Kingdom, recent changes to the NHS GP contract (under the Unplanned Admissions Enhanced Service section), require a minimum of 2% of the risk-identified population to be proactively case managed [16]. In the USA, a number of health insurers and health maintenance organisations offer case management to patients with long-term conditions, for example the ‘Guided care’ and similar programmes [6].

It is important that the provision of case management in primary care should be based on rigorous evidence. While many descriptive reviews exist examining specific types of case management in primary care (such as nurse-led case management [17]), there is no published systematic review of a range of current case management models for high risk individuals in primary care that provides a formal meta-analytic review of its effectiveness across a range of relevant outcomes.

### Objectives

1. To synthesise the evidence for the effectiveness of case management in primary care for ‘at risk’ patients
2. To explore whether the effectiveness of case management in primary care is moderated by the particular model of case management implemented, context, and study design.

## Methods

The methods and results for this review are reported in line with the PRISMA (Preferred Reporting Items for Systematic Reviews and Meta-Analyses) guidelines. The review protocol is available as part of the PROSPERO database (registration number: CRD42014010824).

### Eligibility criteria

Studies were included in this review if they met the following criteria:

- Population: Adults (18+) with long-term condition(s)
  (While prevalence of multimorbidity (i.e. ‘complex’ cases) is highest in the elderly, the absolute numbers affected are greater in those below 65 [18])
- Intervention:
  - Adopting methods to identify ‘at-risk’ patients to receive the case management, with the aim of preventing acute exacerbations of symptoms, and/or secondary care utilisation among those at higher risk
  - Case management, including all of the following activities: case-finding; assessment; care planning; care co-ordination; regular review, monitoring and adaptation of the care plan
  - Primary care/community-based management (regardless of where the case was first identified)
- Comparison: usual care or no-case management
- Outcome categories: **Health**–*self-assessed health status*, *mortality*; **Cost**–*total cost of care*, healthcare *utilisation* (*primary and non-specialist care* and *secondary care* separately), and; **Satisfaction**–*patient satisfaction*
- Study design: Quantitative empirical research, meeting Cochrane Effective Practice and Organisation of Care (EPOC) Group study design criteria: randomised controlled trials (RCTs), non-randomised controlled trials (nRCTs), controlled before and after studies (CBA), and interrupted time series (ITS)

Exclusion criteria:

- Case management targeted solely at care for patients with mental health problems, although mental health conditions could be included where they were co-morbidities alongside physical long-term conditions
- Hospital discharge planning (short-term management to facilitate the transition from hospital to home [19])
- Non-English language papers and grey literature

### Search Strategy

Six main electronic bibliographic databases were searched for potential studies from inception until end of April 2014: MEDLINE (Ovid), EMBASE (Ovid), CINAHL, Cochrane Register of Controlled Trials (CENTRAL), Health Management Information Consortium (Ovid), and CAB Global Health (Ovid),. The search strategy used three key blocks of terms (including subject headings as well as text-words): 1) Case management2) EPOC methodology filter[20]3) Primary care filter[21]. S1 Appendix shows an example of the full search strategy for the MEDLINE database.

Hand searches of the reference lists of included papers, plus previous relevant systematic reviews [17, 22–30] supplemented the database searches.

Results from the above searches were combined in an Endnote library, and duplicates were removed (n = 2186) prior to study selection.

### Study Selection

Study selection was carried out in two stages. First, titles and abstracts of the identified studies were screened in full by the first author. A proportion of these titles and abstracts (10%) were then independently screened by a second author (kappa coefficient = 0.78). Following this initial screening, the full texts of the identified articles were retrieved, and reviewed against the inclusion/exclusion criteria. Forty percent (n = 106/266) of the full text screening was carried out by two reviewers independently. Inter-rater reliability was high (kappa coefficient = 0.81), and any disagreements (n = 7) were resolved by group discussion (resulting in 4 included, and 3 excluded). The remaining full text screening was completed by the first author alone.

### Data extraction

A data extraction form was formulated using Microsoft Excel. The form was initially piloted on two randomly selected studies. The following descriptive data were extracted for included studies:

- Patient: target population; total sample size (intervention/control); proportion of males; average age; average baseline number of long-term conditions; average baseline number of emergency department visits/ hospital admissions in previous year
- Intervention: name of the case management model; brief description of model; intensity of intervention; multidisciplinary team(and specific members) or single case manager; primary case manager; primary location of case management; risk stratification model used; whether there was 24-hour availability of a case manager; caseload; whether the case manager received training in the intervention protocol; reimbursement method
- Context: country was used to define the ‘strength’ of primary health care, classified according to Starfield & Shi’s work [31]
- Outcome categories: **Health**–*self-assessed health status*, *mortality*; **Cost**–*total cost of care*, healthcare *utilisation* (*primary and non-specialist care* and *secondary care* separately), and; **Satisfaction**–*patient satisfaction*
- Study design: design; study duration; unit of analysis; eligibility criteria; type of control group

On a separate sheet, relevant quantitative data for the meta-analysis were extracted (see quantitative analysis section below). Where adjusted and unadjusted results were both presented, the result adjusting for the most potentially confounding variables was extracted.

25 percent (n = 9 studies) of the data were extracted by two researchers working independently. The agreement was high (kappa coefficient = 0.85, across 326 data points), and the remainder of the data were extracted by the first author, and the accuracy of extraction verified by a second reviewer.

### Quality Assessment

In the original protocol, we predicted having to use multiple measures of risk of bias to suit the various study types included in the eligibility criteria. However, having identified the full text articles and study designs represented, it became clear that it would be possible and preferable to use a single quality assessment tool, the EPOC risk of bias tool [32], better allowing comparison of quality across the included studies. The EPOC risk of bias tool encompasses nine standardised criteria to judge the quality of all RCTs, nRCTs, CBA and ITS studies. Each of the nine criteria is judged on a 3-point scale, corresponding to: low risk, unclear risk, and high risk. To ease comparison between studies, the total number of criteria met by each included study was also reported. Those studies at high risk of bias (fulfilling three or less criteria) were removed from the synthesis for sensitivity analysis.

### Quantitative Analysis

Meta-analysis was carried out on six outcome categories related to the three main health system goals [5]. These were: **Health**–*self-assessed health status*, *mortality*; **Cost**–*total cost of care*, healthcare *utilisation* (*primary and non-specialist care* and *secondary care* separately), and; **Satisfaction**–*patient satisfaction*. Table 1 clarifies which measures were included within each of these outcome categories.

**Table 1:** Outcome measures.

**Self-assessed health status**	**Mortality**
- (Instrumental/) Activities of Daily Living	- Mortality within study period
- Physical/ mental health questionnaires	
- Bed days/ restricted activity days	
- Quality Adjusted Life Years (QALYs)	
**Total cost of services**	**Utilisation of primary and non-specialist care**
- Total cost	- Primary care physician visits
- Total insurance expenditure/ reimbursement	- Home care visits
	- Social worker visits
	- Nursing visits
**Utilisation of secondary care**	**Patient satisfaction**
- Emergency Department visits	- Patient satisfaction questionnaires
- Hospital admissions/ re-admissions/ days	- Patient quality of care ratings
- Inpatient/outpatient utilisation	
- Skilled nursing facility visits/ days	
- Ambulance calls	

In addition to the outcomes specified in the original protocol, we also attempted to extract data related to the outcome category of ‘patient safety’: *admissions for ambulatory care sensitive conditions* [33], and *polypharmacy* (simple count of medications). However, none of the included studies reported these outcome measures, so results could not be synthesised.

Meta-analysis was carried out on each outcome, distinguishing between effects over the short-term (0–12 months), and longer-term (13+ months). Meta-analysis used the standardised mean difference measure, based on the mean of the case management group minus mean of the control group, divided by the pooled standard deviation [34]. When multiple measures were available for a single study within a certain outcome category, the median effect was used, as recommended in the literature [35] (e.g. for the outcome of *self-assessed health status*, if a measure of activities of daily living, of restricted activity days, and a measure of QALYs were all available for a given study, the effect size for each of these would be calculated, and the *median* standardised mean difference would represent this studies’ overall effect for this outcome). We adopted Cohen’s rule of thumb for interpreting effect sizes, i.e. that 0.2 indicates a small effect, 0.5 a medium, and 0.8 a large effect [36].

Heterogeneity in the outcomes was assessed using the I^2^ statistic, interpreted as the percentage of total variation in the study estimates due to heterogeneity [37]. A random effects model was chosen to present the pooled effect results based on the relatively high level of heterogeneity assumed between studies evaluating a complex intervention in a variety of service contexts.

Funnel plots were performed to assess small sample bias (which may bean indicator of publication bias), but only for those outcomes drawing on 10 or more studies, as recommended [38]. Egger’s test of small-study effects was additionally performed to quantify observations in the funnel plots [39].

As a complex intervention, context may be of some importance when assessing case management [40]. Subgroup analyses were performed where 10 or more studies contributed effect size data. The pre-specified variables were:

- Context: strength of primary health care orientation of the health system (low versus intermediate/ high)
- Type of case management: multidisciplinary team (MDT) versus single case manager; type of risk tool used (judgement versus threshold/ predictive risk modelling); inclusion of a social worker in the case management (versus absence)
- Study design: RCT versus non-RCT

Table 2 discusses the justifications for these choices of subgroup.

**Table 2:** Subgroup analyses.

**Strength of primary care orientation**: ‘Case management’ may be replacing some of the functions of well-co-ordinated, person-centred primary care [12]. The effects of case management may therefore be greater when it is delivered in contexts where routine primary care services are less well developed. To test this hypothesis, we stratified results by the assessed orientation to primary care of the study country’s health system. The primary care orientation scores were developed by Starfield & Shi, and take into account—for each country—both characteristics of health system policy that are conducive to primary care, as well as characteristics of clinical practice [31].
**Multidisciplinary team versus single case manager**: The hypothesis that teams are more effective than individuals at problem solving and delivering services is established across a number of diverse organisational settings [41], and teams have also been advocated in the treatment of patients with long-term conditions [42]. We tested whether case management by teams was more effective than by individuals.
**Type of risk tool used**: Targeting the ‘correct’ patients will be vital to any effective case management programme, particularly when assessed on cost and utilisation outcomes [43]. To test whether identification of the ‘correct’ patients was more effective when carried out by a rule-based model, we compared clinical judgement with rule-based and predictive models.
**Inclusion of a social worker in case management**: Collaboration between health and social services is thought to be important for effective case management [6], particularly of multimorbid patients who frequently have a complex mix of health and social care issues [44]. It also provides an additional, ‘professional’ level of care integration to the intervention [45], encouraging the different disciplines to work more closely together. To test the relative effectiveness of inclusion of a social worker, we therefore stratified results by this variable.
**RCT versus non-RCT**: RCTs are theoretically less vulnerable to bias, and therefore may give slightly different estimates of effect compared to observational studies (smaller/larger/reversed) [46]. We therefore compared RCTs to non-RCTs to observe any potential inconsistencies.

Statistical significance between subgroups was judged by overlap of each subgroup’s pooled effect (i.e. overlap of confidence intervals between subgroup effects indicates no significant difference) [47].

The majority of effect sizes were calculated using the Metaeasy software add-in for Microsoft Excel (version 1.0.4) [35]. The Metaeasy software allows standardisation of effect size from a variety of input parameters (dichotomous, continuous or both data types), according to eight possible methods described by the Cochrane Collaboration [48]. When multiple methods are available for a single outcome, methods are prioritised according to expected statistical precision [35]. To maximise included results, the Metaeasy effect size calculation methods were supplemented by methods developed by Lipsey & Wilson [49], with a calculator available at http://www.campbellcollaboration.org/resources/effect_size_input.php. Effect directions were transformed so that a positive effect represented favouring case management for all outcome measures. These final effect sizes and their standard errors were then input to STATA together with relevant study information for the subgroup analysis. The final meta-analyses were then run on STATA (version 13) [50] using the *metan* command [51]. Funnel plots were prepared using the *metafunnel* command [52], and the Egger test with the *metabias* command [53].

### Sensitivity analyses and Multiple comparisons

Two separate post-hoc sensitivity analyses were conducted in addition to the specified PROSPERO protocol. Studies were removed from analysis if they were:

1. at high risk of bias (meeting 3 or less of the criteria for assessment of study quality)
2. set in a Veteran’s health setting, where over 90% of the patients were males.

With multiple comparisons, the chances of inflating type I errors is increased [54]. We therefore used the Holm-Bonferroni adjustment [55] for multiple comparisons to identify potential false positive results.

## Results

**Fig 2:**
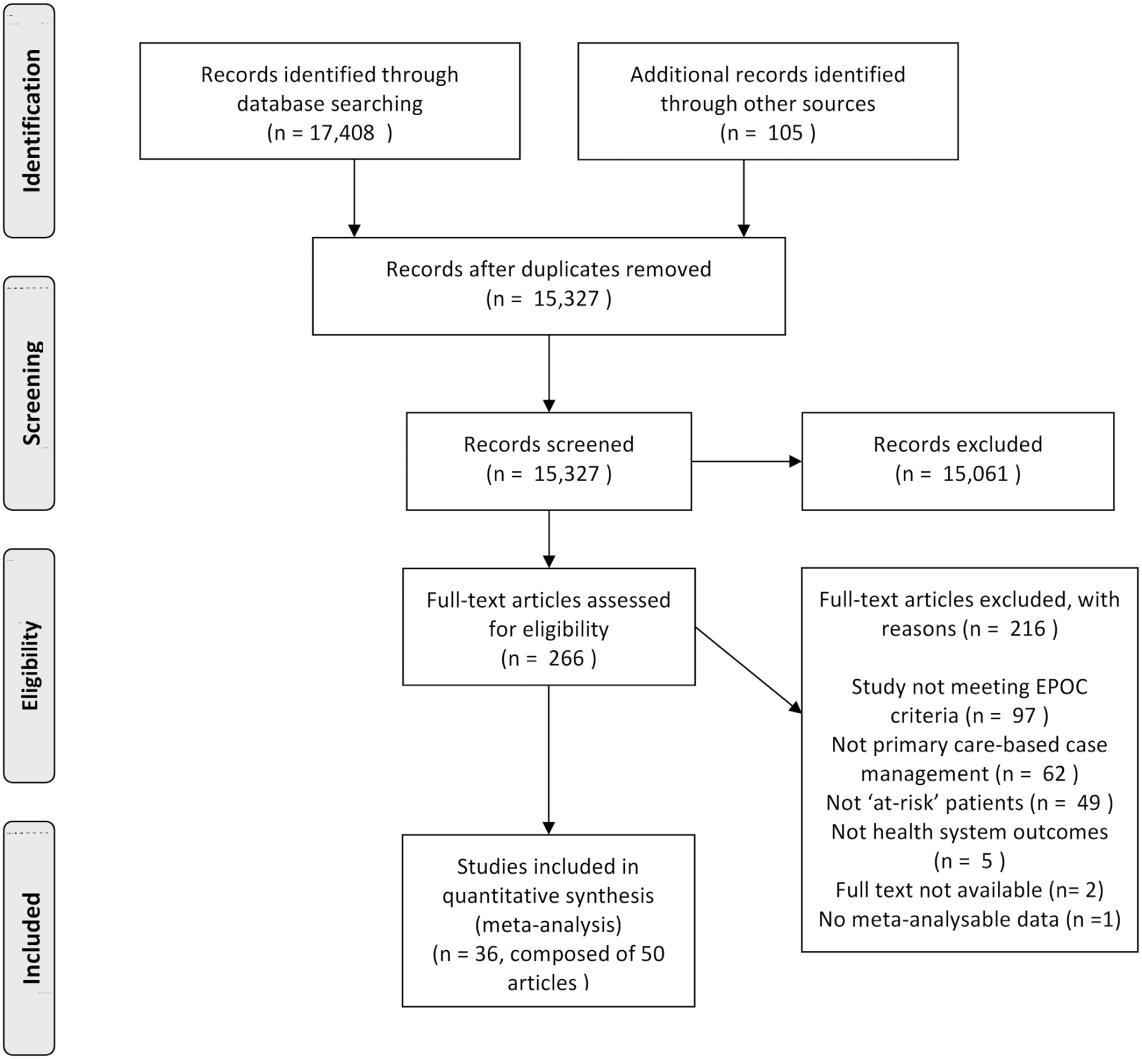
PRISMA flow diagram of study selection [56].

Fig 2 shows the PRISMA flow diagram, with the studies included/excluded at each stage of the screening process. 36 unique studies were finally included in the meta-analyses.

### Characteristics of included studies

Unsurprisingly, since the majority aimed at an elderly population, the average age in nearly all studies was high (mean age: 75.7, range of mean age: 49.0 to 87.3).

Table 3 shows the demographic characteristics of the studies. Of note, 5 (14%) provided case management to a population composed of over 90% males, carried out in veterans’ settings. Inadequate information was provided across studies on baseline number of long-term conditions, and baseline utilisation of emergency and specialist services.

**Table 3:** Demographics of included studies. N/R = Not Reported; N/A = Not Applicable.

Study	Total (n)	Intervention (n)	Control (n)	% Male (controls)	Average age (controls) +-SD	Average no. of chronic conditions (controls) +-SD	Baseline average ED visits in previous year (controls)	Baseline average Hospital admissions in previous year (controls)
**Beland 2006a[57]**; Beland 2006b[58]	1309	656	653	28	82.3+-7.2	5.0+-2.3	N/R	N/R
**Bernabei 1998[59]**	199	99	100	29	81.3+-7.4	4.8+-1.7	N/R	N/R
**Bird 2010[60]**	COPD: 124; CHF: 89	COPD: 78; CHF: 67	COPD: 46; CHF: 22	COPD: 67; CHF: 63	COPD: 70+-N/R; CHF: 76+-N/R	N/R	COPD: 4.8+-3.0; CHF: 5.1+-1.8	COPD: 3.3+-2.1; CHF: 2.8+-1.4
**Boult 2008[61]**; Leff 2009[62]; Boyd 2010[63]; Boult 2011[64]; Boult 2013 [65]	904	485	419	45	78.1+-N/R	4.3+-N/R	N/R	N/R
**Boyd 1996[66]**	54	27	27	30	81+-N/R	N/R	1.1+-N/R	1.6+-N/R
**Burns 1995[67]**; Burns 2000[68]	128	60	68	99	70.8+-3.7	2.0+-1.8	N/R	N/R
**Coburn 2012[69]**	1736	873	863	40	74.9+-6.5	3.8+-2.0	N/R	N/R
**Counsell 2007[70]**; Counsell 2009[71]	951	474	477	23	71.6+-5.8	2.6+-1.5	1.2+-2.4	0.4+-1.2
**Dalby 2000[72]**	142	73	69	23	78.1+-5.3	N/R	N/R	N/R
**De Stampa 2014[73]**	428	105	323	28	87.3+-7.3	N/R	N/R	N/R
**Dorr 2008[74]**	3432	1144	2288	35	76.2+-7.1	N/R	N/R	N/R
**Enguidanos 2006[75]**	452	TCM: 113; GCM: 117; POS: 124	98	36	N/R (65+)	N/R	N/R	N/R
**Fan 2012[76]**	426	209	217	96	65.8+-8.2	N/R	2.7+-2.2	N/R
**Fitzgerald 1994[77]**	668	333	335	100	64.6+-7.7	N/R	N/R	N/R
**Fordyce 1997[78]**	1090	326	764	45	N/R (65+)	N/R	N/R	0.24+-0.4
**Gagnon 1999[79]**	427	212	215	41	81.8+-6.7	N/R	0.9+-1.2	0.4+-0.7
**Gravelle 2007[80]**	7695 (practices)	62 (practices)	6960 (practices)	N/A	N/A	N/A	N/A	N/A
**Hogg 2009[81]**; Gray 2010[82]	241	120	121	37	72.8+-N/R	2.4+-N/R	N/R	N/R
**Kruse 2010[83]**	379	130	249	35	75.1+-6.8	N/R	N/R	N/R
**Leung 2004[84]**	260	130	130	52	75.3+-7.2	2.9+-1.5	0.3+-0.6	0.9+-1.2
**Levine 2012[85]**	298	156	142	36	80.6+-8.7	2.4+-1.5	N/R	N/R
**Martin 2004[86]**	93	44	49	65	69.1+-20	N/R	N/R	N/R
**Metzelthin 2013[87]**	346	153	193	31	76.8+-4.92	N/R	N/R	N/R
**Morishita 1998[88]**; Boult 2001[89]	568	294	274	58	78.7+-5.8	N/R	N/R	0.8+-1.0
**Newcomer 2004[90]**	3079	1537	1542	40	N/R (65+)	N/R	N/R	N/R
**Ploeg 2010[91]**	719	361	358	46	81.3+-4.4	N/R	N/R	N/R
**Rodenas 2008[92]**	152	101	51	N/R	N/R (65+)	N/R	N/R	N/R
**Rubenstein 2007[93]**	793	380	412	97	74.3+-6.1	N/R	N/R	N/R
**Schraeder 2001[94]**	941	530	411	25	75.4+-6.4	N/R	N/R	1.6+-0.94
**Schraeder 2008[95]**	677	400	277	40	76.4+-7.9	N/R	N/R	N/R
**Shannon 2006[96]**; Alkema 2007[97]	781	377	404	34	83.7+-7.36	N/R	0.51+-1.06	N/R
**Sledge2006[98]**	96	47	49	41	49+-N/R	N/R	N/R	N/R
**Stuck 2000[99]**	791	264	527	29	81.5+-4.5	N/R	N/R	N/R
**Sylvia 2008[100]**; Boyd 2008[101]	127	62	65	54	75.8+-N/R	2.9+-N/R	N/R	N/R
**Toseland 1996[102]**; Toseland 1997[103]; Engelhardt 1996[104]; Engelhardt 2006 [105]	160	80	80	100	72.6+-5.75	2.6+-1.3	N/R	N/R
**van Hout 2010[106]**	651	331	320	31	81.5+-4.3	2.0+-1.4	N/R	1.6+-3.8

Table 4 summarises the potentially relevant contextual factors. Of the 36 studies included, the majority were from the USA (n = 21, 58%). When classified according to relative strength of primary care orientation, 23 studies (64%) were set in a system with low strength of primary care, and 13 (36%) in an intermediate, or high strength system. Three studies (8%) were targeted at patients with specific conditions (COPD/chronic heart failure) while the majority targeted populations more broadly on frailty, chronic illness or high utilisation (92%).

**Table 4:** Context of included studies.

Study	Country	Strength of primary care orientation (of country)*	Population	Study design (n participants)	Study length (months)	Brief description of model	Extracted outcomes for meta-analysis
**Beland 2006a [57]**; Beland 2006b [58]	Canada	intermediate	Elderly & functionally disabled	RCT; n = 1309	22	Community-based MDTs with full clinical responsibility for delivering and coordinating services. 24-hour availability via phone. Actively followed patients through care trajectory.	Utilisation (primary/secondary care)
**Bernabei 1998 [59]**	Italy	high#	Elderly & receiving home health services/assistance	RCT; n = 199	12	MDT-designed care plan following assessment by GP/case manager. Case manager followed-up every two months, and constantly available to deal with problems and monitor provision of services.	Mortality, Self-reported health status, Utilisation (primary/secondary care)
**Bird 2010 [60]**	Australia	intermediate	Frequent presenters for COPD/CHF	CBA; n = 124 (COPD)/n = 89 (CHF)	11	Patients allocated to disease-specific stream based on presentations. Results of initial case facilitator assessment discussed at case conference with MDT. Education, self-management, and coordination focus. Follow-up mostly at home	Mortality, Utilisation (secondary care)
**Boult 2008 [61]**; Leff 2009 [62]; Boyd 2010 [63]; Boult 2011 [64]; Boult 2013 [65]	USA	low	Elderly & high-risk multimorbid	cRCT; n = 904	32	Nurse responsible for assessing, planning care, monitoring, coaching self-management, coordination of services, and education for patient and family. Helped by team of physicians.	Total cost of services, Mortality, Patient satisfaction, Self-reported health status, Utilisation (primary/secondary care)
**Boyd 1996 [66]**	USA	low	Elderly & chronically ill	nRCT; n = 54	12	Community-based, integrating case management in patient’s everyday life, with case manager available to monitor the patient’s chronic illness(es). Developing care plan, coordinating services, and providing counselling support.	Mortality
**Burns 1995 [67]**; Burns 2000 [68]	USA	low	Frail elderly	RCT; n = 98	24	Consistent involvement of MDT (GEM team). Initially assess patient and provide ongoing management. Most appropriate team member for given patient served as main liaison.	Mortality, Self-reported health status, Utilisation (primary/secondary care)
**Coburn 2012 [69]**	USA	low	Elderly & chronically ill	RCT; n = 1736	60	Patients risk-stratified within intervention. Regardless of strata, nurse developed an individualised care plan. Group interventions were also provided by the care managers. Nurses collaborated with other healthcare professionals when required.	Mortality
**Counsell 2007 [70]**; Counsell 2009 [71]	USA	low	Low income elderly	RCT; n = 951	24	Care plan developed in collaboration with MDT. Weekly team meetings to review team successes and problem-solve barriers to implementation. At least monthly home-based care management supported by an electronic medical record and web-based tracking system.	Total cost of services, Mortality, Patient satisfaction, Self-reported health status, Utilisation (secondary care)
**Dalby 2000 [72]**	Canada	intermediate	Frail elderly living in the community	RCT; n = 142	14	Nurse-led comprehensive assessment. Care plan developed in conjunction with primary physician. Follow-up visits and calls as needed. Nurse coordinates further community services	Mortality, Utilisation (primary/secondary care)
**De Stampa 2014 [73]**	France	low	Frail elderly	CBA; n = 428	12	Two-person team responsible for patient’s care trajectory. The primary care manager developed care plan, ongoing role of physician to collaborate and share information. Support as needed from geriatricians.	Self-reported health status, Utilisation (secondary care)
**Dorr 2008 [74]**	USA	low	Elderly & chronically ill	nRCT; n = 3432	24	Case management aimed at addressing social, cognitive, and functional needs. Assisted by specialised IT software including structured protocols and guidelines. Co-creation of care plan with patients.	Mortality, Utilisation (secondary care)
**Enguidanos 2006 [75]**	USA	low	Frail elderly	RCT; n = 452	12	Study compares 4 strategies of care. Telephone case management (single case manager); Geriatric care management (GCM) (MDT involvement in care plan); GCM with purchase of service capability (addition of $2000 of designated paid services within first 6 months); Information and referral assistance (most basic, acts as control group).	Utilisation (primary/secondary care)
**Fan 2012 [76]**	USA	low	Frequent presenters for COPD	RCT; n = 426	12	Initial individual educational programme, needs assessment, and an overview of COPD. Reinforced during group session, and with follow-up phone calls. Individualised plan for flare-ups, including prescriptions for prednisone and antibiotic.	Mortality, Patient satisfaction, Self-reported health status, Utilisation (secondary care)
**Fitzgerald 1994 [77]**	USA	low	Inpatient medical service users	RCT; n = 668	12	Included instructing patients about their medical problems, facilitating access to usual care, and identifying and fulfilling unmet social and medical needs with standard or alternative sources of care. Periodic assessment of medical and social needs. Coordination of all appointments for patient. 24-hour telephone access	Mortality, Utilisation (primary/secondary care)
**Fordyce 1997 [78]**	USA	low	Frail elderly	RCT; n = 1090	36	Yearly health, functional, and social evaluation. Weekly team meetings where nurse presented cases for review. Medical-functioning profile worked up for each patient, acting as indication of intensity of follow-up, as needed. Follow-up mostly by telephone.	Utilisation (secondary care)
**Gagnon 1999 [79]**	Canada	intermediate	Frail elderly	RCT; n = 427	10	Coordination of all healthcare providers and implementation of a responsive plan of care. Monthly phone calls, and a home visit every 6 weeks were the minimum standard. Additional contacts when required. Specialist consultation available to nurses for complicated cases.	Patient satisfaction, Self-reported health status, Utilisation (secondary care)
**Gravelle 2007 [80]**	UK	high	Frail elderly	CBA; n = 7757 (practices)	48	Assessment, using structured assessment tools, a physical examination, which resulted in an individualised care plan. Patients were then monitored at a frequency determined by their classification of risk.	Mortality, Utilisation (secondary care)
**Hogg 2009 [81]**; Gray 2010 [82]	Canada	intermediate	Older & at-risk of adverse outcomes	RCT; n = 241	18	Nurses and pharmacist co-located at family practice, but delivered care almost exclusively at patient’s home. Team-developed care plan. 22 patients also received a tele-health system for remote monitoring.	Total cost of services, Self-reported health status, Utilisation (primary/secondary care)
**Kruse 2010 [83]**	USA	low	Elderly & chronically ill, at-risk for catastrophic illness	nRCT; n = 379	60	Assessed patient’s needs, provided education, coordinated referrals, provided first-access care and follow-up care following visits to doctor/hospital on the telephone.	Mortality, Utilisation (primary/secondary care)
**Leung 2004 [84]**	Hong Kong	intermediate^	Community-dwelling frail elderly	RCT; n = 260	6	Regular home-visits and telephone consultations. Care plan designed in discussion with patient and caregiver. Coordination of health and social services through referral plus case conference. Monitoring of health and hospitalisation patterns via computer programme. Counselling, health education, and supportive group services.	Self-reported health status, Utilisation (primary/secondary care)
**Levine 2012 [85]**	USA	low	Elderly & multimorbid, at-risk for hospitalisation	RCT; n = 298	12	Included early identification and treatment of illness exacerbation, patient-specific health education, self or caregiver management of disease, and advance care planning and other psychosocial issues. Team worked closely at all stages.	Total cost of services, Patient satisfaction, Utilisation (primary/secondary care)
**Martin 2004 [86]**	New Zealand	intermediate+	Acutely deteriorating COPD patients	RCT; n = 93	12	Generic care plan was individualised and signed off. Supplies of antibiotics and prednisone made available. Copies of plan held by each potential provider of care. Routine support and further education available.	Utilisation (primary/secondary care)
**Metzelthin 2013[87]**	The Netherlands	high	Frail elderly	cRCT; n = 346	24	Core team (GP and nurse) cooperate closely with other health professionals as needed. Initial home-visit and assessment, meeting to design care plan, and treatment starts with protocol offering recommendations and guidelines.	Self-reported health status
**Morishita 1998 [88]**; Boult 2001 [89]	USA	low	Elderly & high-risk	RCT; n = 568	18	Consistent involvement of MDT (GEM team). Specialised GEM clinic introduced, where patients were followed-up. Individual team members saw patients approximately monthly, met to discuss. Regular telephone calls, and available 24-hours on telephone service	Total cost of services, Mortality, Patient satisfaction, Self-reported health status, Utilisation (primary care)
**Newcomer 2004 [90]**	USA	low	High-risk elderly	RCT; n = 3079	12	Patients triaged by risk category after initial assessment. Predominant method of contact was telephone, supplemented by monitoring utilisation. Nurse case manager distributed educational material and advice, coordinated services, but no direct role in treatment management.	Self-reported health status, Utilisation (primary/secondary care)
**Ploeg 2010 [91]**	Canada	intermediate	Elderly & at-risk of functional decline	RCT; n = 719	12	Nurse-led comprehensive initial assessment, collaborative care planning, health promotion, and referral to community health and social support services. Assessments at baseline, 6 and 12 months. Additional health education and referrals to other health services.	Total cost of services, Mortality, Self-reported health status, Utilisation (primary/secondary care)
**Rodenas 2008 [92]**	Spain	high	Elderly & receiving home care	RCT; n = 152	12	Direct interaction with the patients was carried out by a MDT. The team took charge of: 1) assessing individual needs 2) designing and starting individual care itineraries 3) benefit quality assurance, and 4) monitoring and on-going review of the strategy. Extra health and social care resources were also available for the intervention group.	Patient satisfaction, Utilisation (primary/secondary care)
**Rubenstein 2007 [93]**	USA	low	High-risk elderly	RCT; n = 793	36	Initial telephone assessment by physician assistant case manager. Some patients referred for further assessment and an interdisciplinary care plan at a geriatric assessment unit. Coordination of follow-up by phone, each patient mailed a copy of the care plan.	Self-reported health status, Utilisation (secondary care)
**Schraeder 2001 [94]**	USA	low	Community-dwelling elderly	RCT; n = 941	24	Team's goal was to provide enhanced primary care by providing assessments, flexible home office visits, detailed care planning, routine telephone monitoring, and coordination and procurement of supportive services. Nurse and care assistant co-located.	Total cost of services, Mortality, Utilisation (secondary care)
**Schraeder 2008 [95]**	USA	low	Community-dwelling, chronically ill elderly	nRCT; n = 677	36	Intervention emphasised collaboration between physicians, nurses and patients, risk identification, comprehensive assessment, collaborative planning, health monitoring, patient education, and transitional care. Nurse and care assistant co-located.	Utilisation (secondary care)
**Shannon 2006 [96]**; Alkema 2007 [97]	USA	low	Elderly & high utilisers	RCT; n = 781	12	Telephone-based management to coordinate services bridging medical and social care. Focus on referrals. Monthly follow-up calls.	Mortality, Utilisation (primary/secondary care)
**Sledge 2006 [98]**	USA	low	Recent high use of inpatient services	RCT; n = 96	12	PIC intervention consisted of two components: 1) a comprehensive interdisciplinary medical and psychosocial assessment (2–3 hours on first visit), and 2) follow-up ambulatory case management for 1 year. Involvement differed by need, but minimum monthly call.	Total cost of services, Mortality, Patient satisfaction, Self-reported health status, Utilisation (primary/secondary care)
**Stuck 2000 [99]**	Switzerland	low#	In-home visits for disability prevention	RCT; n = 791	36	Annual nurse-led comprehensive assessments. Cases discussed with geriatrician and recommendations developed. In-home follow-up visits every 3 months. Nurses also provided health education, encouraged self-care, and attempted to improve communication with the physician. Interdisciplinary team available to discuss complex patients.	Mortality, Self-reported health status, Utilisation (secondary care)
**Sylvia 2008 [100]**; Boyd 2008 [101]	USA	low	Community-dwelling, chronically ill, elderly	nRCT; n = 127	6	At-home assessment, evidence-based care plan, promotion of self-management, monthly monitoring, coaching on healthy behaviours, coordination of transitions in care, and facilitating access to community resources.	Total cost of services, Patient satisfaction, Utilisation (primary/secondary care)
**Toseland 1996 [102]**; Toseland 1997 [103]; Engelhardt 1996 [104]; Engelhardt 2006 [105]	USA	low	Frail elderly	RCT; n = 160	48	Primary functions of the GEM team included: initial comprehensive assessment; development of a care plan; implementation of the care plan; periodic reassessment; monitoring and updating the care plan, and; referral to and coordination with other health and social service providers. Weekly team meetings to discuss.	Total cost of services, Mortality, Patient satisfaction, Self-reported health status, Utilisation (primary/secondary care)
**van Hout 2010 [106]**	The Netherlands	high	Community-dwelling frail elderly	RCT; n = 651	18	Assessment of health and care needs, recommended interventions based on guidelines, individually tailored care plans (copy left at patient’s home for other care workers to see/add to). Home visits at least 4 times a year.	Mortality, Self-reported health status, Utilisation (secondary care)

* Source: Starfield et al 2002 [31], unless otherwise stated

^#^ Source: Macinko et al 2003 [107]

^+^ Source: Grant et al 1997 [108]

^^^ Source: Fry & Horder 1994 [109]

A brief qualitative description of each intervention is also provided in Table 4. Table 5 compares some of the key attributes of each intervention more directly. Many criteria highlighted as key to understanding integrated care interventions [9] were inadequately reported which limited their utility for analysis. However, the type of risk tool, whether the case management was carried out by a MDT or single case manager, and the inclusion of a social worker in the case management could be recorded for all studies. The majority of studies used a ‘threshold’/’predictive risk modelling’ risk assessment tool (n = 32, 89%), with only 4 (11%) using clinical judgement. Twenty-one studies (58%) employed MDT case management. A social worker was involved in the case management in 12 studies (33%).

**Table 5:** Details of interventions.

Study	Name of case management model	Intensity of intervention (patient contacts)	Risk Assessment Tool (judgement/threshold/predictive risk modelling)	MDT or single case manager (primary case manager in bold)	Primary location of case management	24-hour availability of case manager	Caseload per manager/ team	Training received by case manager	Case management reimbursement method
**Beland 2006a [57]**; Beland 2006b [58]	SIPA [French acronym for System of Integrated Care for Older Persons]	Not clear	**Threshold** *Functional Autonomy Measurement System (SMAF)*	MDT: **nurse/social worker**, community nurses; occupational therapists, homemakers, staff family physicians, (consultant pharmacists), (community organisers)	Not clear	Yes	35–45	Yes	Family physician offered $400 per SIPA patient in addition to their usual FFS
**Bernabei 1998 [59]**	Integrated community care	Every 2 months	**Threshold** *previous use of home services*	MDT: **trained case manager**, general practitioner, geriatrician, social worker, nurses	Not clear	Not clear	Not clear	Yes	Not clear
**Bird 2010 [60]**	HARP [Hospital Admission Risk Programme]	4–7 times in 12 months	**Threshold** *previous hospital use*	MDT: **trained case facilitator**, N/S	Home	Not clear	Not clear	Unclear	Not clear
**Boult 2008 [61]**; Leff 2009 [62]; Boyd 2010 [63]; Boult 2011 [64]; Boult 2013 [65]	Guided care	Monthly	**Predictive risk modelling** *Hierarchical Condition Category (HCC)*	MDT: **Nurse**, physicians	Not clear	Not clear	50–60	Yes	FFS
**Boyd 1996 [66]**	Community-based case management	Averaged 4.45 hours per patient per month	**Threshold** *previous secondary care use*	Single: **nurse**	Home	Not clear	Not clear	Unclear	Not clear
**Burns 1995 [67]**; Burns 2000 [68]	GEM [Geriatric Evaluation and Management]	Not clear	**Threshold** *mixture of criteria judging frailty*	MDT: **GEM team** (physician, nurse, social worker, psychologist)	GEM clinic	Not clear	Not clear	Yes	Not clear
**Coburn 2012 [69]**	Community-based nursing intervention	Minimum of monthly. Average 17.4 contacts per patient per year	**Predictive risk modelling** *Sutter Health Questionnaire/numeric risk score*	Single: **nurse**	Various	Not clear	85–110	Yes	FFS + fixed fee per participant per month
**Counsell 2007 [70]**; Counsell 2009 [71]	GRACE [Geriatric Resources for Assessment and Care of Elders]	Minimum of monthly	**Threshold** *income level*	MDT: **nurse/social worker**, geriatrician, pharmacist, physical therapist, mental health social worker, community-based services liaison	Home/ telephone	Not clear	Not clear	Unclear	Not clear
**Dalby 2000 [72]**	Visiting nurse	Not clear	**Threshold** *Questionnaire (functional impairment/past hospital use)*	Single: **nurse**	Home	Not clear	Not clear	Unclear	Capitation
**De Stampa 2014 [73]**	COPA [CO-ordinationPersonnesAgées]	Not clear	**Threshold** *Contact Assessment (CA) tool*	MDT: **Nurse**, primary care physician, (geriatrician)	Home	Not clear	40	Yes	Not clear
**Dorr 2008 [74]**	CMP [Care Management Plus]	Not clear	**Judgement** *clinical judgement*	Single: **nurse**	Not clear	Not clear	Not clear	Yes	Not clear
**Enguidanos 2006 [75]**	Kaiser Permanente Community Partners	TCM: 4–5 contacts per patient per 4-week period GCM: Approx 20 hours per case over 8–9 months	**Threshold** *functional/utilisation criteria*	TCM- Single: **social worker** GCM- MDT: **nurse/social worker**, geriatrician, assistant department manager	TCM: Telephone GCM: Home/ telephone	Not clear	Not clear	Unclear	Not clear
**Fan 2012 [76]**	CCMP [Comprehensive Care Management Program]	Monthly for 3 months. Every 3 months thereafter.	**Threshold** *previous hospital use*	Single: **healthcare professional**(qualification varied by site)	Telephone	No	Not clear	Yes	Not clear
**Fitzgerald 1994 [77]**	GMC [General Medicine Clinic] case management	Averaged 1.6 per patient per month	**Threshold** *previous hospital use*	Single: **nurse**	Clinic/ Telephone	Yes	Not clear	Unclear	Salaried nurse
**Fordyce 1997 [78]**	STAR [Senior Team Assessment and Referral programme]	Not clear	**Threshold** *STAR questionnaire (measuring frailty)*	MDT: **nurse**, geriatrician, health educator, geriatric psychiatrist	Telephone	Not clear	Not clear	Unclear	Not clear
**Gagnon 1999 [79]**	Community-based nurse case management	Minimum monthly call, and home visit every 6 weeks.	**Predictive risk modelling** *Boult assessment tool (40% or more probability of hospitalisation)*	Single: **nurse**	Home/ telephone	No	40–55	Yes	Not clear
**Gravelle 2007 [80]**	Evercare	Not clear	**Threshold** *previous emergency admissions*	Single: **nurse**	Not clear	Not clear	Not clear	Unclear	Not clear
**Hogg 2009 [81]**; Gray 2010 [82]	APTCare [Anticipatory and Preventive Team Care]	Not clear	**Judgement** *clinical judgement*	MDT: **nurse**, pharmacist, usual family physician	Home	Not clear	Not clear	Yes	FFS/ capitation
**Kruse 2010 [83]**	Nurse care coordination	Not clear	**Threshold** *previous outpatient use*	Single: **nurse**	Clinic/ telephone	Not clear	Not clear	Unclear	Not clear
**Leung 2004 [84]**	Case Management Project	Once every two weeks.	**Threshold** *previous hospital use*	MDT: **nurse/social worker**, geriatricians, senior social workers, geriatric nursing specialist, clinical psychologist, rehabilitation therapists	Home/ telephone	Not clear	Not clear	Unclear	Not clear
**Levine 2012 [85]**	CHA [Choices for Healthy Aging]	Minimum monthly	**Predictive risk modelling** *electronic risk assessment tool*	MDT: **team** (physician, nurse practitioner, nurse care manager, social worker)	Home/ telephone	Yes	Not clear	Unclear	Not clear
**Martin 2004 [86]**	Care plans for COPD	Visits at 0, 3, 6, and 12 months.	**Threshold** *previous COPD exacerbations requiring care*	MDT: **nurse**, respiratory specialist, GP	Not clear	Not clear	Not clear	Unclear	Not clear
**Metzelthin 2013[87]**	PoC [Prevention of Care]	Not clear	**Threshold** *Groningen Frailty Indicator*	MDT: **nurse**, GP, (occupational therapist), (physical therapist), (other health professionals as needed)	Home	Not clear	Not clear	Yes	Not clear
**Morishita 1998 [88]**; Boult 2001 [89]	GEM [Geriatric Evaluation and Management]	Monthly clinic visits + telephone availability	**Predictive risk modelling** *probability of repeated admission instrument*	MDT: **GEM team** (geriatrician, geriatric nurse practitioner, nurse, social worker)	GEM clinic/ telephone	Yes	Not clear	Unclear	FFS
**Newcomer 2004 [90]**	ECM [Enhanced Case Management]	Minimum monthly. Weekly until problem resolution. Average 7.7 hours per patient over 12 months.	**Threshold** *presence of chronic conditions (subsequently stratified by risk score obtained from assessment questionnaire)*	Single: **nurse**	Telephone	Not clear	250 (~60 actively case managed at any time)	Unclear	Not clear
**Ploeg 2010 [91]**	Preventive primary care outreach	Minimum 3 yearly visits + follow-up phone calls/home visits.	**Threshold** *Sherbrooke postal questionnaire (assessing risk of functional decline)*	Single: **nurse**	Home/ telephone	Not clear	Not clear	Unclear	Capitation-based that includes some FFS
**Rodenas 2008 [92]**	Case management Valencia	Minimum once every 2 months.	**Judgement** *referral protocol of social and health cases*	MDT: **team** (physician, nurse, social worker)	Not clear	Not clear	Not clear	Yes	Not clear
**Rubenstein 2007 [93]**	Screening, case finding, referral	One month after first contact. Every 3 months thereafter.	Threshold *Geriatric Postal Screening Survey*	Single **Physician assistant**	Telephone	Not clear	Not clear	Unclear	Not clear
**Schraeder 2001 [94]**	Collaborative primary care nurse case management	Average 8 contacts per patient per year.	**Judgement/ Threshold** *clinical judgement/presence of determined risk factors*	MDT: **nurse/case assistant**, primary care physician	Various	Not clear	Not clear	Unclear	Not clear
**Schraeder 2008 [95]**	Collaborative primary care nurse case management	Minimum monthly	**Threshold** *health screening questionnaire*	MDT: **nurse**, case assistant, primary care physician	Various	Not clear	Not clear	Unclear	Not clear
**Shannon 2006 [96]**; Alkema 2007 [97]	Care Advocate Programme	Minimum monthly	**Predictive risk modelling** *health care utilisation algorithm*	Single: **social worker**	Telephone	Not clear	Not clear	Unclear	Not clear
**Sledge 2006 [98]**	PIC [Primary Intensive Care]	Minimum monthly	**Threshold** *previous hospital use*	MDT: **psychiatric nurse**, social worker, psychiatrist, general internist	Telephone	Not clear	21	Unclear	Not clear
**Stuck 2000 [99]**	In-home visits for disability prevention	Every 3 months.	**Threshold** *Scoring on 6 criteria generated from the literature*	Single: **nurse**	Home	Not clear	Not clear	Yes	Not clear
**Sylvia 2008 [100]**; Boyd 2008 [101]	Guided care	Minimum monthly	**Predictive risk modelling** *Adjusted Clinical Groups Predictive Model*	MDT: **nurse**, primary care physician	Not clear	Not clear	50–60	Yes	Capitated insurance system
**Toseland 1996 [102]**; Toseland 1997 [103]; Engelhardt 1996 [104]; Engelhardt 2006 [105]	GEM [Geriatric Evaluation and Management]	Not clear	**Threshold** *previous outpatient use + functional impairments*	MDT: **nurse**, geriatrician, social worker	GEM clinic	Not clear	Not clear	Unclear	Not clear
**van Hout 2010 [106]**	Nurse home visits	Minimum 4 visits per patient per year	**Threshold** *frailty score (COOP-WONCA charts)*	Single: **nurse**	Home	Not clear	Not clear	Yes	Not clear

### Methodological Quality

The majority of studies (n = 28, 78%) used an RCT. The length of follow-up in the studies varied, with a range of 6 to 60 months. Table 6 shows the methodological quality according to the nine criteria of the EPOC risk of bias tool. The studies were of variable quality, with 64% (n = 23) fulfilling seven or more criteria, 30% (n = 11) fulfilling between four and six criteria, and 6% (n = 2) fulfilling three or less.

**Table 6:** Quality of included studies.

Study	Was the allocation sequence adequately generated?	Was the allocation adequately concealed?	Were baseline outcome measurements similar?	Were baseline characteristics similar?	Were incomplete outcome data adequately addressed?	Was knowledge of the allocated interventions adequately prevented during the study?	Was the study adequately protected against contamination?	Was the study free from selective outcome reporting?	Was the study free from other risks of bias?	Criteria met
**Beland 2006a [57]**; Beland 2006b [58]	Yes	Yes	Yes	Yes	Yes	Yes	Yes	Yes	Yes	**9**
**Bernabei 1998 [59]**	Yes	Yes	Yes	Yes	Yes	Yes	Yes	Yes	Yes	**9**
**Bird 2010 [60]**	No	No	No	No	Yes	Yes	Yes	Yes	No	**4**
**Boult 2008 [61]**; Leff 2009 [62]; Boyd 2010 [63]; Boult 2011 [64]; Boult 2013 [65]	Yes	Yes	Yes	Yes	Yes	Yes	Yes	Yes	Yes	**9**
**Boyd 1996 [66]**	No	Unclear	Yes	Yes	Unclear	Yes	Unclear	Yes	Unclear	**4**
**Burns 1995 [67]**; Burns 2000 [68]	Yes	Yes	Yes	Yes	Unclear	Yes	Yes	Yes	Yes	**8**
**Coburn 2012 [69]**	Yes	Yes	Yes	Yes	Yes	Yes	Yes	Yes	Yes	**9**
**Counsell 2007 [70]**; Counsell 2009 [71]	Yes	Yes	Yes	Yes	Yes	Yes	Yes	Yes	Yes	**9**
**Dalby 2000 [72]**	Yes	Yes	Yes	Yes	Yes	Yes	Yes	Unclear	Yes	**8**
**De Stampa 2014 [73]**	No	No	Yes	Yes	Yes	Yes	Yes	Yes	Yes	**7**
**Dorr 2008 [74]**	No	Yes	Yes	Yes	Yes	Yes	Yes	Yes	No	**7**
**Enguidanos 2006 [75]**	Unclear	Unclear	Yes	Yes	Unclear	Unclear	Unclear	Unclear	Yes	**3**
**Fan 2012 [76]**	Yes	Yes	Yes	Yes	Yes	Yes	Unclear	Yes	Yes	**8**
**Fitzgerald 1994 [77]**	Unclear	Yes	Yes	Yes	Yes	Yes	Yes	Yes	Yes	**8**
**Fordyce 1997 [78]**	Unclear	Unclear	No	No	Yes	Yes	Unclear	Yes	Yes	**4**
**Gagnon 1999 [79]**	Yes	Yes	Yes	Yes	Yes	Yes	No	Yes	Yes	**8**
**Gravelle 2007 [80]**	No	No	Yes	No	Unclear	Yes	Yes	Yes	Yes	**5**
**Hogg 2009 [81]**; Gray 2010 [82]	Yes	Yes	Yes	Yes	Yes	Yes	Yes	Yes	Yes	**9**
**Kruse 2010 [83]**	No	Yes	Unclear	No	Yes	Yes	Yes	Yes	Yes	**6**
**Leung 2004 [84]**	Unclear	Unclear	No	Yes	Unclear	Yes	Unclear	Yes	Yes	**4**
**Levine 2012 [85]**	Yes	Unclear	Unclear	Yes	Yes	Yes	Unclear	Yes	Yes	**6**
**Martin 2004 [86]**	Unclear	Unclear	Unclear	No	Unclear	Yes	Unclear	Yes	Yes	**3**
**Metzelthin 2013[87]**	Yes	Yes	Yes	Yes	Yes	Yes	Yes	Yes	Yes	**9**
**Morishita 1998 [88]**; Boult 2001 [89]	Yes	Yes	Yes	Yes	Yes	Yes	Yes	Yes	Unclear	**8**
**Newcomer 2004 [90]**	Unclear	Unclear	Yes	Yes	Yes	Yes	Unclear	Yes	Yes	**6**
**Ploeg 2010 [91]**	Yes	Yes	Unclear	Yes	Yes	Yes	Yes	Yes	Yes	**8**
**Rodenas 2008 [92]**	Yes	Yes	No	No	Yes	Yes	Unclear	Yes	Yes	**6**
**Rubenstein 2007 [93]**	Yes	Yes	Yes	Unclear	No	Yes	Yes	Yes	Yes	**7**
**Schraeder 2001 [94]**	Unclear	Yes	No	No	Yes	Yes	Yes	Yes	Yes	**6**
**Schraeder 2008 [95]**	No	Yes	Yes	Yes	Yes	Yes	Yes	Yes	Yes	**8**
**Shannon 2006 [96]**; Alkema 2007 [97]	Unclear	Yes	Yes	Yes	Yes	Yes	Yes	Yes	Yes	**8**
**Sledge 2006 [98]**	Yes	Yes	Unclear	Yes	Yes	Yes	Yes	Yes	Yes	**8**
**Stuck 2000 [99]**	Yes	Yes	Yes	Yes	Yes	Yes	Yes	Yes	Yes	**9**
**Sylvia 2008 [100]**; Boyd 2008 [101]	No	Yes	Yes	Yes	Yes	Yes	Unclear	Yes	Yes	**7**
**Toseland 1996 [102]**; Toseland 1997 [103]; Engelhardt 1996 [104]; Engelhardt 2006 [105]	Unclear	Unclear	Unclear	Yes	Yes	Yes	Unclear	Yes	Yes	**5**
**van Hout 2010 [106]**	Yes	Yes	Unclear	Yes	Yes	Yes	Yes	Yes	Yes	**8**

### Primary analyses

**Fig 3:**
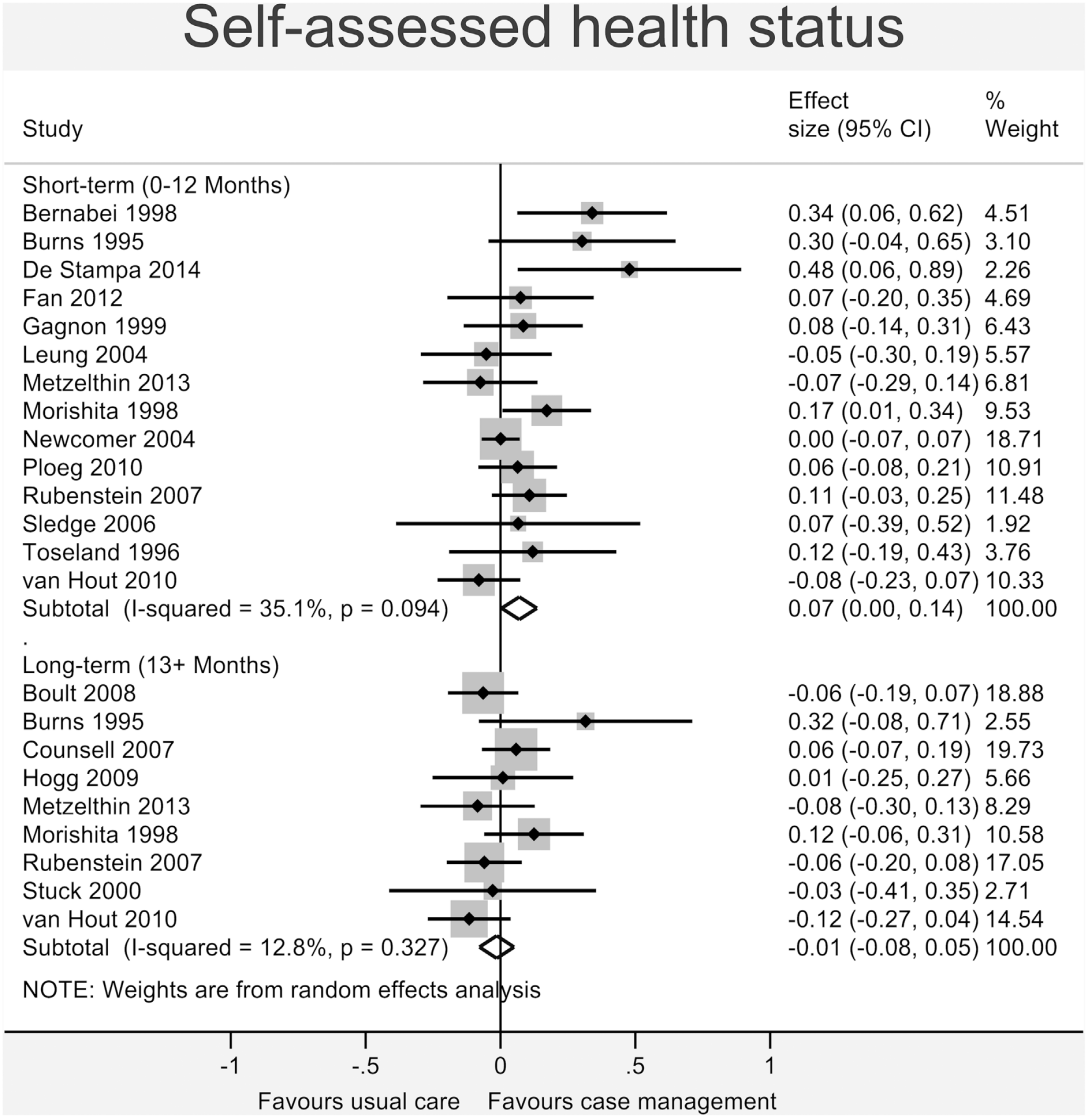
Forrest plot for self-assessed health status outcome. Effect estimates are the standardised mean difference, where the solid vertical line at 0 indicates no effect. Effect estimates are based on a random-effects model. Each subtotal shows the overall effect estimate for the time-period indicated.

**Fig 4:**
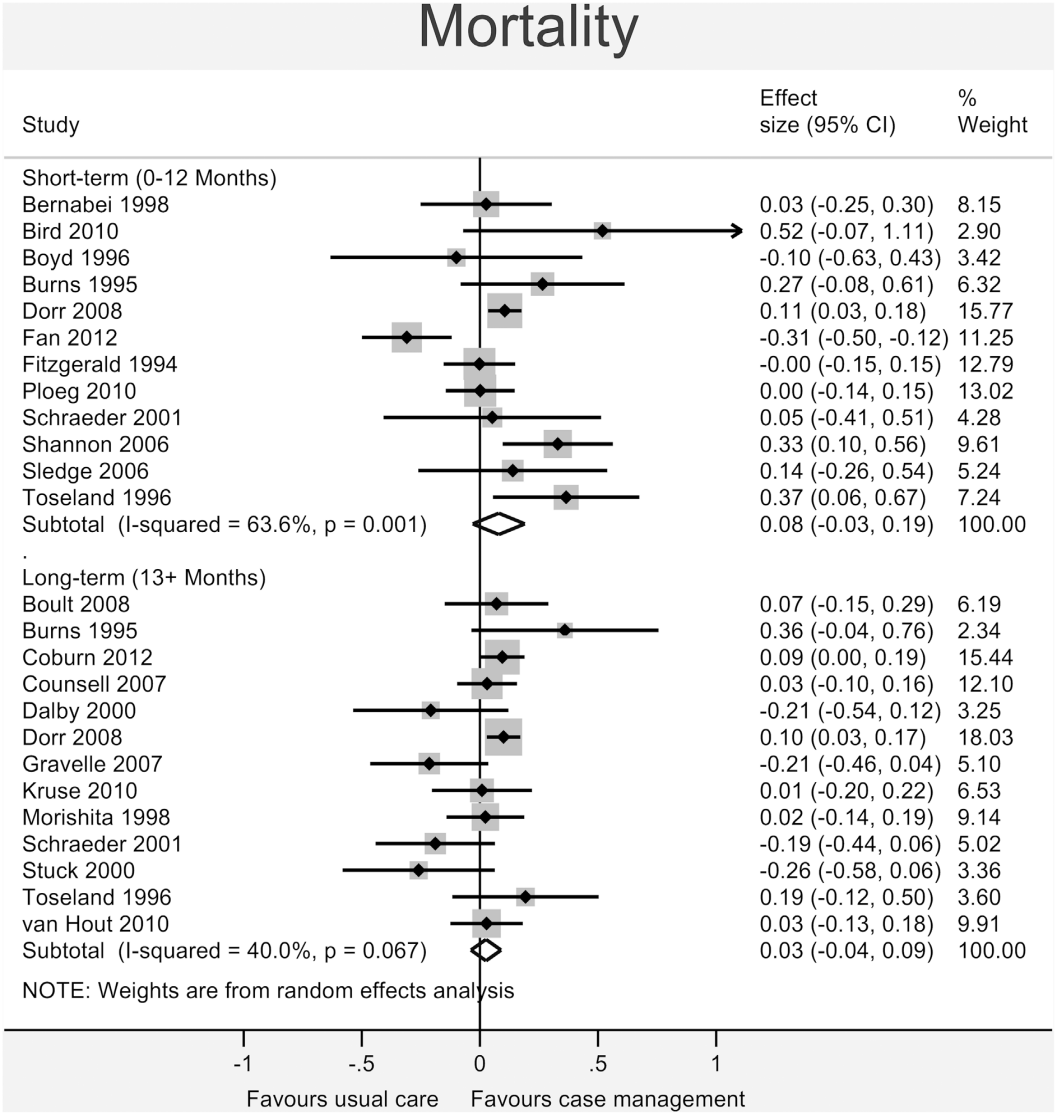
Forrest plot for mortality outcome. Effect estimates are the standardised mean difference, where the solid vertical line at 0 indicates no effect. Effect estimates are based on a random-effects model. Each subtotal shows the overall effect estimate for the time-period indicated.

**Fig 5:**
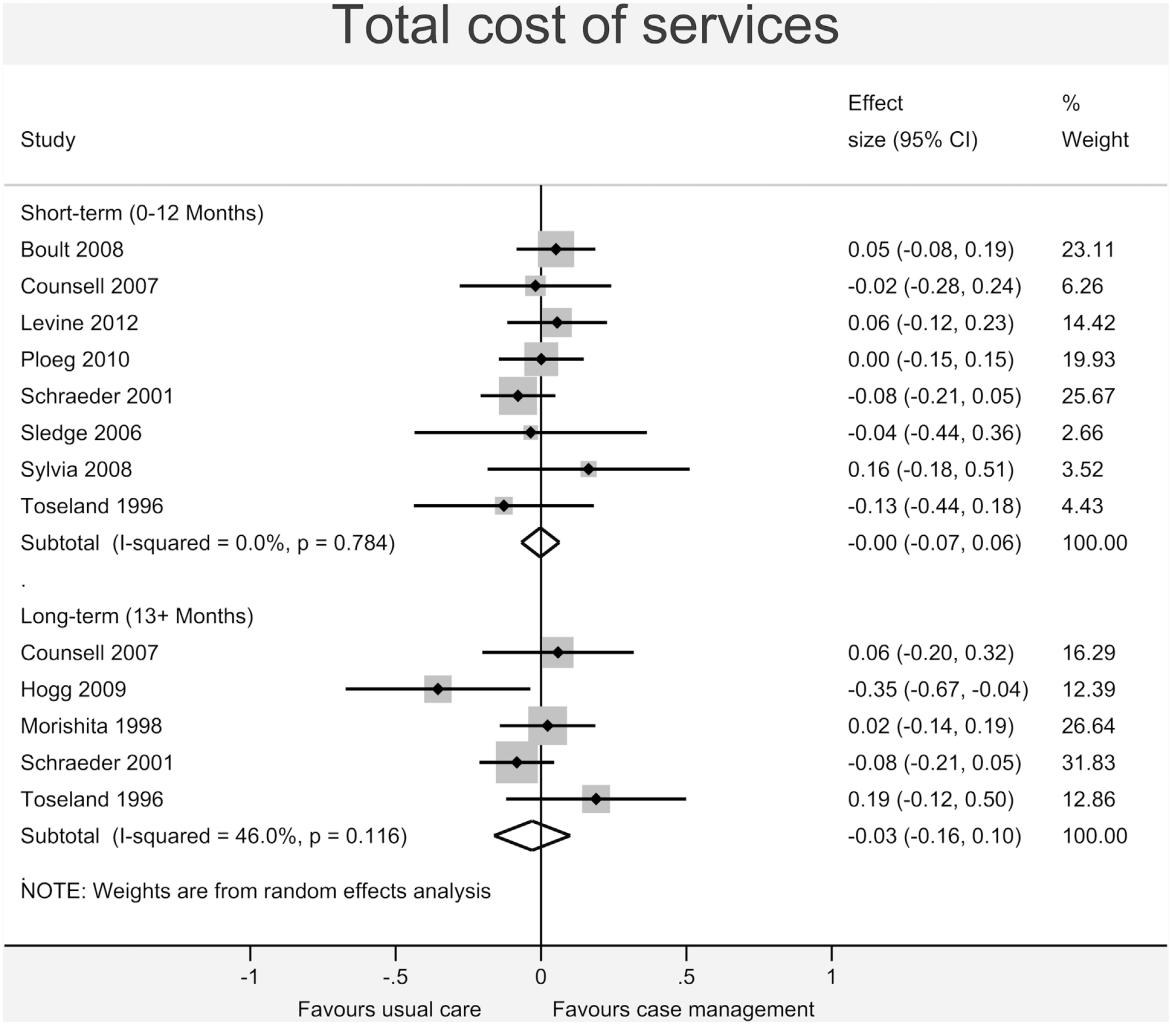
Forrest plot for total cost of services outcome. Effect estimates are the standardised mean difference, where the solid vertical line at 0 indicates no effect. Effect estimates are based on a random-effects model. Each subtotal shows the overall effect estimate for the time-period indicated.

**Fig 6:**
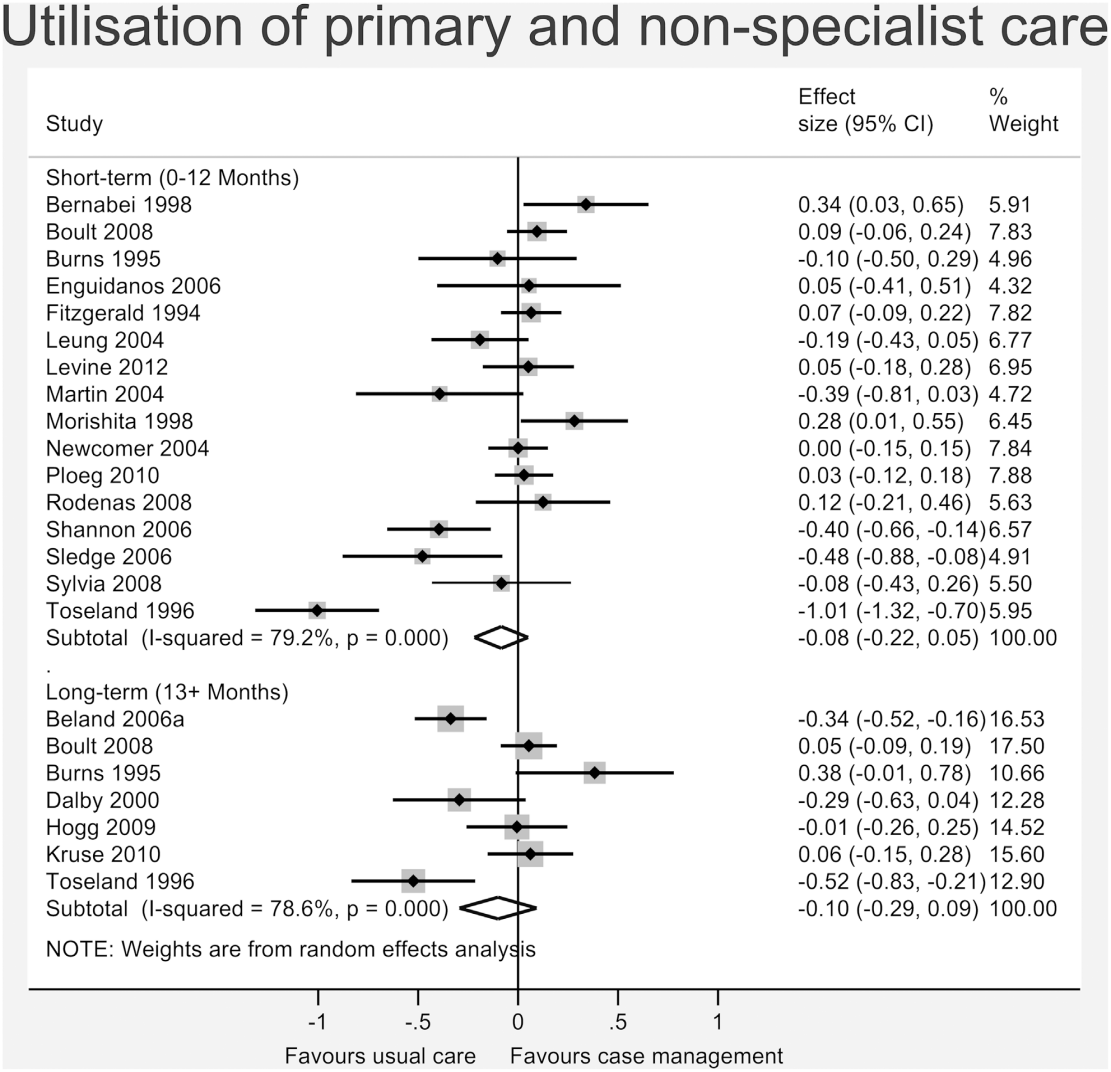
Forrest plot for utilisation of primary and non-specialist care outcome. Effect estimates are the standardised mean difference, where the solid vertical line at 0 indicates no effect. Effect estimates are based on a random-effects model. Each subtotal shows the overall effect estimate for the time-period indicated.

**Fig 7:**
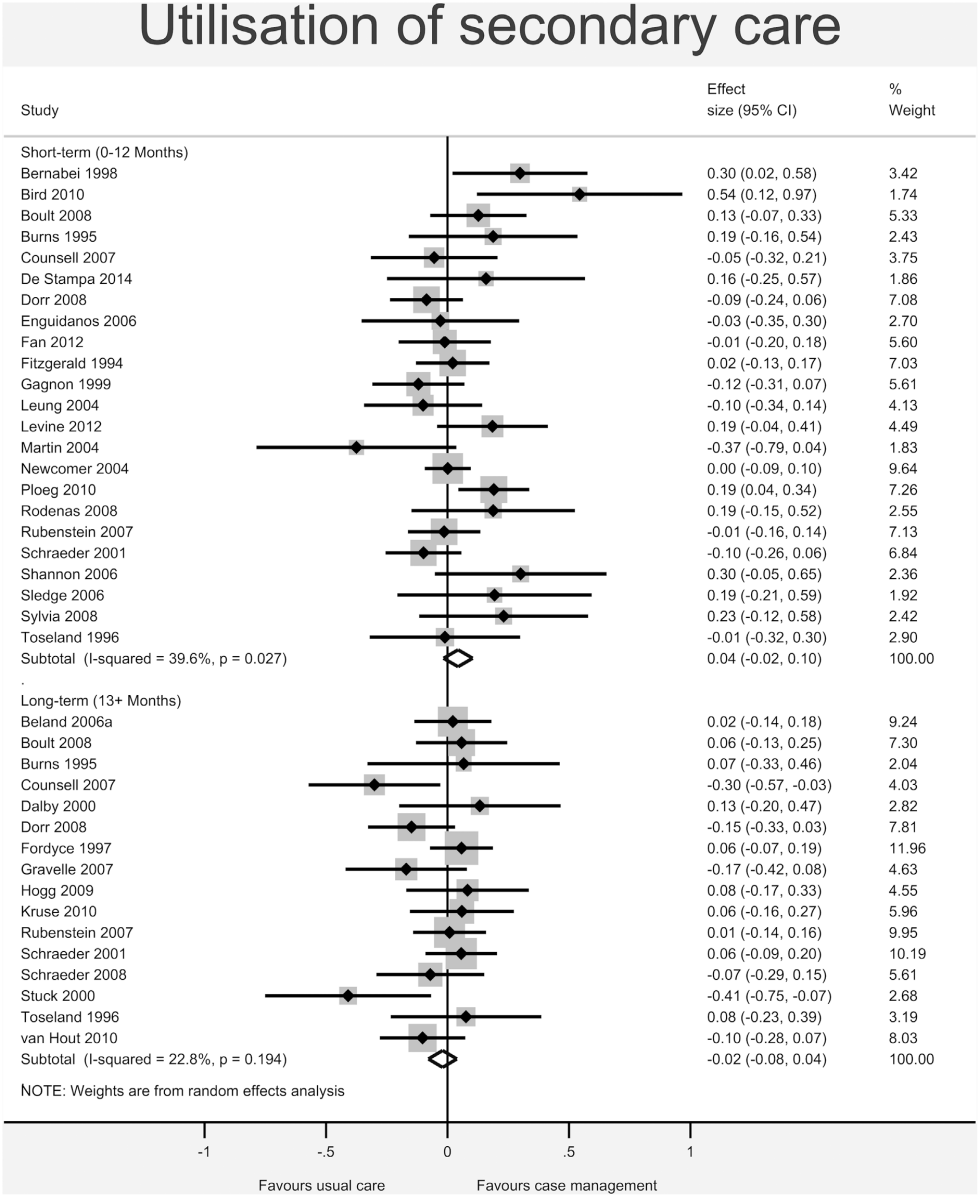
Forrest plot for utilisation of secondary care outcome. Effect estimates are the standardised mean difference, where the solid vertical line at 0 indicates no effect. Effect estimates are based on a random-effects model. Each subtotal shows the overall effect estimate for the time-period indicated.

**Fig 8:**
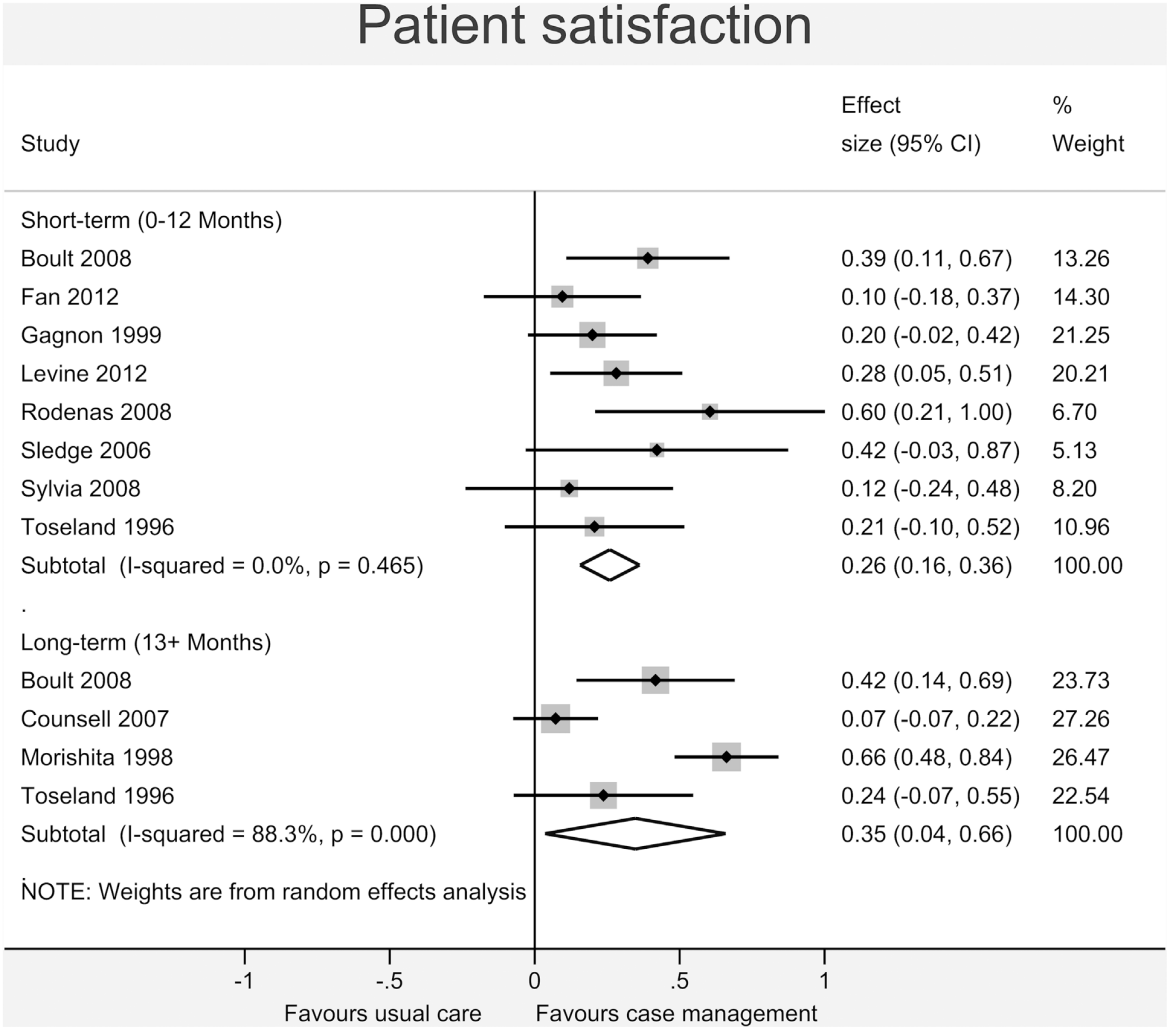
Forrest plot for patient satisfaction outcome. Effect estimates are the standardised mean difference, where the solid vertical line at 0 indicates no effect. Effect estimates are based on a random-effects model. Each subtotal shows the overall effect estimate for the time-period indicated.

Figs 3–8 show the results of the primary meta-analyses for the six outcome categories assessed (both short- and long-term).

#### Health

A statistically significant effect favouring case management was found for *self-assessed health status* (Fig 3) in the short-term (0.07, 95% CI 0.00 to 0.14, I^2^ = 35.1%, p = 0.094), but this effect was not present in the long-term (-0.01, 95% CI -0.08 to 0.05, I^2^ = 12.8%, p = 0.327). No significant effect was found for *mortality* (short-term: 0.08, 95% CI -0.03 to 0.19, I^2^ = 63.6%, p = 0.001; long-term: 0.03, 95% CI -0.04 to 0.09, I^2^ = 40.0%, p = 0.067 –Fig 4).

#### Cost

No significant effect was found for *total cost of services* (short-term: -0.00, 95% CI -0.07 to 0.06, I^2^ = 0.0%, p = 0.784; long-term: -0.03, 95% CI -0.16 to 0.10, I^2^ = 46.0%, p = 0.116 –Fig 5), *utilisation of primary and non-specialist care* (short-term: -0.08, 95% CI -0.22 to 0.05, I^2^ = 79.2%, p<0.001; long-term: -0.10, 95% CI -0.29 to 0.09, I^2^ = 78.6%, p<0.001 –Fig 6) or *secondary care* (short-term: 0.04, 95% CI -0.02 to 0.10, I^2^ = 39.6%, p = 0.027; long-term: -0.02, 95% CI -0.08 to 0.04, I^2^ = 22.8%, p = 0.194 –Fig 7).

#### Satisfaction

*Patient satisfaction* (Fig 8) showed a statistically significant beneficial effect in the case management group in the short-term (0.26, 95% CI 0.16 to 0.36, I^2^ = 0.0%, p = 0.465), increasing in the long-term (0.35, 95% CI 0.04 to 0.66, I^2^ = 88.3%, p<0.001).

Heterogeneity, measured with the I^2^ statistic, varied by outcome and time-period measured. Those with particularly high I^2^ (over 75% [47]), included *utilisation of primary and non-specialist care* (short- and long-term), and *patient satisfaction* (long-term).

The funnel plots showed a fairly even distribution of small studies, suggesting no small study bias. The one exception was for *self-assessed health status*, which appeared slightly skewed towards favourable results for the intervention in smaller studies. However, results of the Egger test found no statistically significant small-study effects across any of the outcomes assessed.

### Subgroup analyses

The following outcome categories met the minimum criteria of 10 studies contributing to the primary analysis: *mortality* (short-, and long-term), *self-assessed health status* (short-term), *utilisation of primary and non-specialist care* (short-term), and *utilisation of secondary care* (short-, and long-term).

The results for each of the subgroup analyses are summarised in Table 7, below (the forest plots can be found in S2 Appendix).

**Table 7:** Results of subgroup analyses. No significant differences between subgroups (p<0.05). *Note*: Positive effect size favours case management for all measures.

Outcome (time-period)	Subgroup effect size ^(number of studies)^	
	**MDT** ^**(21)**^	**Single** ^**(15)**^
Mortality (short)	0.20 (0.05 to 0.35)* ^**(6)**^	0.01 (-0.13 to 0.16)^**(6)**^
Mortality (long)	0.04 (-0.06 to 0.14)^**(6)**^	0.01 (-0.08 to 0.10)^**(7)**^
Self-rated health (short)	0.14 (0.01 to 0.27)* ^**(8)**^	0.02 (-0.03 to 0.07)^**(6)**^
Utilisation primary care (short)	-0.10 (-0.30 to 0.10)^**(12)**^	-0.04 (-0.20 to 0.11)^**(4)**^
Utilisation secondary care (short)	0.08 (-0.02 to 0.17)^**(15)**^	0.01 (-0.06 to 0.09)^**(8)**^
Utilisation secondary care (long)	0.02 (-0.04 to 0.09)^**(9)**^	-0.08 (-0.18 to 0.03)^**(7)**^
	**Low PHC score** ^**(23)**^	**Int/high PHC score** ^**(13)**^
Mortality (short)	0.09 (-0.05 to 0.23)^**(9)**^	0.05 (-0.13 to 0.23)^**(3)**^
Mortality (long)	0.05 (-0.01 to 0.12)^**(10)**^	-0.10 (-0.27 to 0.08)^**(3)**^
Self-rated health (short)	0.11 (0.02 to 0.20)* ^**(8)**^	0.03 (-0.08 to 0.13)^**(6)**^
Utilisation primary care (short)	-0.12 (-0.30 to 0.06)^**(11)**^	-0.00 (-0.20 to 0.20)^**(5)**^
Utilisation secondary care (short)	0.01 (-0.03 to 0.06)^**(16)**^	0.08 (-0.10 to 0.26)^**(7)**^
Utilisation secondary care (long)	-0.02 (-0.10 to 0.05)^**(11)**^	-0.02 (-0.12 to 0.07)^**(5)**^
	**Clinical Judgement** ^**(4)**^	**Risk modelling** ^**(32)**^
Mortality (short)	0.10 (0.03 to 0.17)* ^**(2)**^	0.09 (-0.06 to 0.24)^**(10)**^
Mortality (long)	-0.02 (-0.30 to 0.26)^**(2)**^	0.02 (-0.05 to 0.09)^**(11)**^
Self-rated health (short)	n/a	n/a
Utilisation primary care (short)	n/a	n/a
Utilisation secondary care (short)	-0.06 (-0.18 to 0.06)^**(3)**^	0.06 (-0.00 to 0.13)^**(20)**^
Utilisation secondary care (long)	-0.01 (-0.15 to 0.14)^**(3)**^	-0.02 (-0.09 to 0.04)^**(13)**^
	**RCT** ^**(28)**^	**Non-RCT** ^**(8)**^
Mortality (short)	0.07 (-0.07 to 0.22)^**(9)**^	0.12 (-0.06 to 0.30)^**(3)**^
Mortality (long)	0.03 (-0.05 to 0.10)^**(10)**^	-0.00 (-0.18 to 0.17)^**(3)**^
Self-rated health (short)	n/a	n/a
Utilisation primary care (short)	n/a	n/a
Utilisation secondary care (short)	0.04 (-0.02 to 0.10)^**(19)**^	0.17 (-0.11 to 0.45)^**(4)**^
Utilisation secondary care (long)	-0.00 (-0.07 to 0.07)^**(12)**^	-0.08 (-0.19 to 0.02)^**(4)**^
	**Social worker** ^**(12)**^	**No social worker** ^**(24)**^
Mortality (short)	0.24 (0.10 to 0.37)* ^**(5)**^	-0.01 (-0.14 to 0.13)^**(7)**^
Mortality (long)	0.07 (-0.04 to 0.17)^**(4)**^	-0.00 (-0.09 to 0.08)^**(9)**^
Self-rated health (short)	0.15 (0.04 to 0.27)* ^**(6)**^	0.03 (-0.04 to 0.10)^**(8)**^
Utilisation primary care (short)	-0.13 (-0.38 to 0.12)^**(10)**^	0.03 (-0.05 to 0.10)^**(6)**^
Utilisation secondary care (short)	0.10 (0.00 to 0.20)^**(10)**^	0.02 (-0.06 to 0.09)^**(13)**^
Utilisation secondary care (long)	-0.04 (-0.21 to 0.13)* ^**(4)**^	-0.02 (-0.08 to 0.05)^**(12)**^

* = significant in-subgroup effect (p<0.05)

Power to determine differences in subgroup analyses is limited, the large number of comparisons risks inflating rates of Type I error, and there may be other differences between studies that have not been taken into account in these univariate comparisons. Therefore, these results should be treated with appropriate caution. When interpreting subgroup effects, significant difference between subgroups is the important comparative factor. Importantly, no statistically significant differences were found when comparing between subgroups.

However, results perhaps indicate slightly beneficial effects of delivery of case management by an MDT, with the inclusion of a social worker, and in settings with low strength of primary care. These preliminary findings may merit further investigation. Nevertheless, any significant within-subgroup effects found were extremely small by Cohen’s interpretation.

### Sensitivity Analysis and Multiple comparisons

Those studies at highest risk of bias reported findings in the short-term (0–12 months) for *utilisation of primary and non-specialist care* and *utilisation of secondary care* [75, 86]. Studies using Veteran participants [67, 76, 77, 93, 102], with over 90% males, reported findings in all outcomes and time-periods assessed.

After adjusting for multiple comparisons, excluding these studies showed no significant difference from the results reported above, either for the primary analysis, or between subgroup differences for the subgroup analyses. The results of the sensitivity analysis can be found in S3 Appendix.

After Holm-Bonferroni correction was applied to all results, only two of the statistically significant results held: the finding of a significant effect on *patient satisfaction* in the short-term (0–12 months) in the primary analysis, and the same outcome measure in the sensitivity analysis (excluding studies with Veteran participants).

## Discussion

### Summary of the key findings

Case management of ‘at-risk’ patients in primary care has been promoted as a way of reducing health system pressures, and the most recent iteration of the UK GP contract has provided incentives for its delivery. This evidence identified by this review does not provide strong evidence to suggest that case management is an effective way of alleviating pressure on a health system. *Total cost of care*, and *utilisation of secondary care* services do not appear to be significantly affected by case management. There may be a significant effect on *self-reported health status* with case management. However, the magnitude of the benefit is very modest, does not meet conventional criteria even for a ‘small’ effect, and was not significant after adjustment for multiple comparisons. Case management does improve *patient satisfaction* when compared to usual care. This is a legitimate outcome for a ‘patient-centred’ health care system, but is rarely seen as the primary aim of case management interventions.

### Strengths and limitations

Strengths of this study include the use of PRISMA guidelines, pre-specification of subgroups, as well as the broad search strategy. Unfortunately, the broad search impaired our ability to double-screen all studies at every stage, although we did double-screen a proportion at every stage, and our inter-rater reliability was consistently good. We did not include grey literature, due to the generally lower quality of this literature [110]. We found no evidence of small study bias in our included studies.

Assessing complex service-level interventions is difficult, and RCTs may be particularly problematic in the context of patients with multimorbidity [111]. We included the range of intervention study types considered by ‘The Cochrane Effective Practice and Organisation of Care (EPOC) Group’.

We view the use of meta-analysis as a major strength of this piece of work, which differentiates this review from the narrative syntheses [17, 22–24, 26–28, 30]. Some argue that meta-analysis of complex service-level interventions is inappropriate, because the effects of the intervention are so dependent on context [40], and pooling the results from different contexts is not advisable. However, as shown in the introduction, case management can be defined in terms of a number of common components. In addition, we did try to account for context differences (such as strength of the primary care system), although the precise scope of the term is unclear [112], and a lack of consistent reporting limited what was possible.

Heterogeneity was high for measures of *utilisation of primary and non-specialist care* in both time-periods assessed, and *patient satisfaction* in the long-term. This high level of heterogeneity is expected in analysis of a complex intervention, which is possibly highly dependent on context. On the whole, choosing a random effects model took into account expected heterogeneity arising from comparison of a complex intervention across different settings [113]. Nevertheless, caution must be applied to uncritical interpretation of the pooled effect, due to the level of unexplained variation observed.

When we adjusted for multiple comparisons, only increased *patient satisfaction* in the short-term remained significant. This type of adjustment, while it reduces the risk of false positive findings (type I error), does so at the risk of inflating the number of false negative findings (type II error) [114]. As an intervention with low risk of harm to the patient, we have chosen to present the unadjusted results as the primary analyses, with the results adjusted for multiple comparisons suggesting additional caution in interpretation.

The outcome measures we chose were broadly inclusive. For example, in *self-assessed health status* we included activities of daily living, as well as bed days, and more typical ‘health’ measures, for instance QALYs. This could be a potential weakness of this study. However, we chose these broad outcome categories attempting to synthesise as much of the relevant data as possible that were reported within the selected studies. Furthermore, these measures were reported as functional outcome measures of health in the individual studies, and were therefore synthesised as such.

Utilisation and cost outcomes have a tendency to be skewed. As expected, the studies we synthesised reporting these outcomes demonstrated significant skew (i.e. the mean is smaller than twice the standard deviation), indicating that the mean reported is not a good indicator of the centre of the distribution [115]. Future primary studies should make sure these skews are reported, and that the effects of any subsequent log transformation are detailed for more precise synthesis of these outcomes. Furthermore, although costs were detailed in a number of studies, we identified only one cost-effectiveness analysis [82], and one cost-benefit analysis [84].

### Interpretation of the results in the context of other studies

It is difficult to directly compare, as most previous reviews on this subject have used narrative synthesis methods [17, 23, 24], or used ‘vote counting’ to quantify the number of studies with statistically significant results in either direction [22, 26–28, 30]. The majority of existing reviews conclude that despite theoretical benefits, in practice there is only slight evidence of benefits [22, 23], particularly related to patient satisfaction [24, 27], and functional health [26]. The single previous systematic review we identified which employed meta-analysis, additionally included hospital discharge planning interventions (identifying a total of eleven studies, only six of which—three in the primary care setting—were included in meta-analysis), and only used meta-analysis for a single outcome category, ‘unplanned hospital admissions’, similarly finding no significant effect [25]. Our results are in line with those previous reviews, with the additional benefits of updating the evidence base, quantifying the impacts (emphasising the *small* benefits) across a range of outcome categories, and exploring contextual variations.

Most published reviews focused on implementation have similarly identified inadequate reporting of the methods of applying case management in practice as a major limitation of the literature [17].

### Implications for research

Case management has potential to impact on patient safety issues in primary care, such as co-ordination and communication between professionals and levels of the health system [116]. No safety outcomes were identified in the included literature, and primary care patient safety is a notoriously under-researched area [117].

As multimorbid patients are likely at most risk for co-ordination failures, and therefore potentially have most to gain from the integration of care, measures of multimorbidity must be more consistently reported [118], even if this is a simple count of mean number of chronic diseases. Ideally, however, this would give more detailed breakdown by disease type/cluster [119] as a subgroup analysis, enabling further targeting of specific interventions to specific groups of patients who are most likely to benefit [120]. Additionally, there is some evidence that the coexistence of physical and mental health problems could lead to increased management difficulties [121]. Comorbidity of conditions should be better reported in evaluations and explored in further research.

Current evidence comes from a majority of high-income, Western settings. This potential bias requires addressing with evidence from other settings, for example Asia, where the case management approach is currently evolving.

### Implications for policy and practice

Given the lack of significant effects across the majority of outcome categories, should case management generally be encouraged or incentivised for the treatment of ‘at-risk’ patients in primary care? This review would suggest that, as currently delivered, case management should not be regarded as a primary means of reducing overall health service utilisation and that it will not reduce costs or improve health outcomes. While we have shown some statistically significant benefits, these are not focused on primary outcomes, with the largest overall effect on satisfaction, which did not meet the usual criterion for a ‘medium’ effect [36]. However, the current results rest on the evidence accumulated from RCTs. There are potential problems associated with this study design in the assessment of complex interventions and conditions [111], although other designs which may be better able to reflect routine delivery of case management (such as controlled before and after designs [80]) have their own problems with internal validity.

Evidence from the subgroup analyses do perhaps point to more effective ways of delivering the intervention, namely: delivery by a MDT as opposed to a single case manager, and the inclusion of a social worker. These findings agree with the wider literature which advocates the use of a multidisciplinary team to successfully manage patients with chronic disease [42], and advocates better integration of health and social care [45]. Case management may be more effective in a system where the strength of primary health care orientation is low. However, these subgroup results should be interpreted with caution, as they are exploratory univariate analyses, which should be investigated further while controlling for potential confounding factors before firm conclusions are drawn. Furthermore, the significance of these effects did not withstand adjustment for multiple comparisons.

Further understanding of factors driving the effectiveness of case management may benefit from on-going evaluation of implementation at the local level. It is important that components of implementation are reported consistently and in detail, so that these can be included in future systematic reviews and effectiveness of individual elements of the intervention can be examined.

## Conclusions

Current evidence suggests case management of ‘at-risk’ patients in primary care is not effective beyond small improvements in patient satisfaction. Case management should not be regarded as a proven technology in the delivery of integrated care, there remains a need for further enhancement and evaluation of its effectiveness, particularly with study designs which better incorporate context, and in lower income settings. More research is needed into more effective methods of delivery (e.g. by an MDT and including a social worker), and implementation (e.g. in a health system with poor primary care orientation), which may additionally improve effectiveness. Even with these improvements, however, case management may never be as effective as it needs to be to deliver major savings through a focus on high risk groups [122]. This highlights the need for a variety of models to deal with system pressures, including integrated care at different levels of the health care system, and with more of focus on the wider population of patients [123].

## Supporting Information

S1 Appendix Full MEDLINE search strategy. (DOCX)

S2 Appendix Forest plots for subgroup analyses. (DOCX)

S3 Appendix Results of the sensitivity analyses. (DOCX)
