# Supplementary material for: Effectiveness of Case Management for 'At Risk' Patients in Primary Care: A Systematic Review and Meta-Analysis
Source: PLoS One. 2015 Jul 17;10(7):e0132340. doi: 10.1371/journal.pone.0132340 (PMC4505905; doi:10.1371/journal.pone.0132340)
Supplement: S1 Appendix — (DOCX) [file pone.0132340.s001.docx]

|  | **Keywords** |
| --- | --- |
| 1 | Family Practice/ |
| 2 | Primary Health Care/ |
| 3 | Physicians, Family/ |
| 4 | Community Health Services/ |
| 5 | Community Dentistry/ |
| 6 | Community Health Nursing/ |
| 7 | Community Mental Health Services/ |
| 8 | Community Pharmacy Services/ |
| 9 | Home Care Services/ |
| 10 | Community Mental Health Centers/ |
| 11 | family pract$.tw. |
| 12 | general practice$.tw. |
| 13 | community based.tw. |
| 14 | community care.tw. |
| 15 | family medicine.tw. |
| 16 | family physician$.tw. |
| 17 | primary care.tw. |
| 18 | (primary health care or primary healthcare).tw. |
| 19 | family doctor$.tw. |
| 20 | primary medical care.tw. |
| 21 | general physician$.tw. |
| 22 | general practitioner$.tw. |
| 23 | primary care practitioner$.tw. |
| 24 | (community adj (health or healthcare or health care)).tw. |
| 25 | primary healthcare team$.tw. |
| 26 | primary health care team$.tw. |
| 27 | primary medical care team$.tw. |
| 28 | practice nurse$.tw. |
| 29 | practice manager$.tw. |
| 30 | (gpsi or gpwsi).tw. |
| 31 | (practitioner$ adj3 special interest$).tw. |
| 32 | (primary care or primary health care or general practice or family practice or family medicine).nw. |
| 33 | or/1-32 |
| 34 | case management.tw. |
| 35 | care management.tw. |
| 36 | care co?ordination.tw. |
| 37 | collaborat* care.tw. |
| 38 | collaborat* practice.tw. |
| 39 | virtual ward*.tw. |
| 40 | care integrat*.tw. |
| 41 | care team?.tw. |
| 42 | co?ordinated care.tw. |
| 43 | multidisciplin* care.tw. |
| 44 | interdisciplin* care.tw. |
| 45 | multidisciplin* team?.tw. |
| 46 | interdisciplin* team?.tw. |
| 47 | multidisciplin* management.tw. |
| 48 | interdisciplin* management.tw. |
| 49 | (integrated adj4 care).tw. |
| 50 | exp Patient Care Team/ |
| 51 | care plan*.tw. |
| 52 | practice team?.tw. |
| 53 | exp Patient Care Planning/ |
| 54 | exp "Delivery of Health Care, Integrated"/ |
| 55 | or/34-54 |
| 56 | randomized controlled trial.pt. |
| 57 | controlled clinical trial.pt. |
| 58 | multicenter study.pt. |
| 59 | (randomis* or randomiz* or randomly allocat* or random allocat*).ti,ab. |
| 60 | groups.ab. |
| 61 | (trial or multicenter or multi center or multicentre or multi centre).ti. |
| 62 | (intervention* or controlled or control group or compare or compared or (before adj5 after) or (pre adj5 post) or pretest or pre test or posttest or post test or quasiexperiment* or quasi experiment* or evaluat* or effect or impact or time series or time point? or repeated measur*).ti,ab. |
| 63 | or/56-62 |
| 64 | exp Animals/ |
| 65 | Humans/ |
| 66 | 64 not (64 and 65) |
| 67 | review.pt. |
| 68 | meta analysis.pt. |
| 69 | news.pt. |
| 70 | comment.pt. |
| 71 | editorial.pt. |
| 72 | cochrane database of systematic reviews.jn. |
| 73 | comment on.cm. |
| 74 | (systematic review or literature review).ti. |
| 75 | or/66-74 |
| 76 | 63 not 75 |
| 77 | 33 and 55 and 76 |
