# Supplementary material for: Effectiveness of Case Management for 'At Risk' Patients in Primary Care: A Systematic Review and Meta-Analysis
Source: PLoS One. 2015 Jul 17;10(7):e0132340. doi: 10.1371/journal.pone.0132340 (PMC4505905; doi:10.1371/journal.pone.0132340)
Supplement: S2 Appendix — (DOCX) [file pone.0132340.s002.docx]

# S2 Appendix: Forest plots for subgroup analyses*

*** No significant results remain following Holm-Bonferroni correction for multiple comparisons**

## **Multidisciplinary team versus single case manager**

## **Strength of primary care orientation**

## **Type of risk tool used**

## **RCT versus non-RCT**

## **Inclusion of a social worker in case management**
