# Supplementary material for: Effectiveness of Case Management for 'At Risk' Patients in Primary Care: A Systematic Review and Meta-Analysis
Source: PLoS One. 2015 Jul 17;10(7):e0132340. doi: 10.1371/journal.pone.0132340 (PMC4505905; doi:10.1371/journal.pone.0132340)
Supplement: S3 Appendix — (DOCX) [file pone.0132340.s003.docx]

# S1 Appendix:

# Results of the sensitivity analysis excluding studies at high risk of bias*

*** No significant results remain following Holm-Bonferroni correction for multiple comparisons**

## Primary analysis – sensitivity analysis

## Secondary analysis – sensitivity analysis

| **Outcome (time-period)** | **Subgroup effect size ^(number of studies)^** | |
| --- | --- | --- |
|  | **MDT ^(19)^** | **Single ^(15)^** |
| Utilisation primary care (short) | -0.09 (-0.31 to 0.14) **^(10)^** | -0.04 (-0.20 to 0.11) **^(4)^** |
| Utilisation secondary care (short) | 0.10 (0.01 to 0.20)* **^(13)^** | 0.01 (-0.06 to 0.09) **^(8)^** |
|  | **Low PHC score ^(22)^** | **Int/high PHC score ^(12)^** |
| Utilisation primary care (short) | -0.14 (-0.33 to 0.05) **^(10)^** | 0.05 (-0.14 to 0.25) **^(4)^** |
| Utilisation secondary care (short) | 0.01 (-0.03 to 0.06) **^(15)^** | 0.13 (-0.05 to 0.31) **^(6)^** |
|  | **Clinical Judgement ^(4)^** | **Risk modelling ^(30)^** |
| Utilisation primary care (short) | n/a | n/a |
| Utilisation secondary care (short) | -0.06 (-0.18 to 0.06) **^(3)^** | 0.07 (0.01 to 0.14)* **^(18)^** |
|  | **RCT ^(26)^** | **Non-RCT ^(8)^** |
| Utilisation primary care (short) | n/a | n/a |
| Utilisation secondary care (short) | 0.04 (-0.02 to 0.10) **^(17)^** | 0.17 (-0.11 to 0.45) **^(4)^** |
|  | **Social worker ^(11)^** | **No social worker ^(23)^** |
| Utilisation primary care (short) | -0.15 (-0.42 to 0.12) **^(9)^** | 0.04 (-0.03 to 0.11) **^(5)^** |
| Utilisation secondary care (short) | 0.11 (0.01 to 0.22)* **^(9)^** | 0.03 (-0.04 to 0.10) **^(12)^** |

No significant difference between subgroups (p<0.05)

* = significant in-subgroup effect (p<0.05)

*Note*: Positive effect size favours case management for all measures

# Results of the sensitivity analysis excluding studies conducted in Veteran’s settings (over 90% male population)*

*** No significant results remain following Holm-Bonferroni correction for multiple comparisons**

## Primary analysis – sensitivity analysis

## Secondary analysis – sensitivity analysis

| **Outcome (time-period)** | **Subgroup effect size ^(number of studies)^** | |
| --- | --- | --- |
|  | **MDT ^(19)^** | **Single ^(12)^** |
| Mortality (short) | n/a | n/a |
| Mortality (long) | 0.01 (-0.08 to 0.10) **^(4)^** | 0.01 (-0.08 to 0.10) **^(7)^** |
| Self-rated health (short) | 0.13 (-0.03 to 0.29) **^(6)^** | 0.00 (-0.05 to 0.06) **^(4)^** |
| Utilisation primary care (short) | 0.01 (-0.14 to 0.15) **^(10)^** | -0.09 (-0.30 to 0.12) **^(3)^** |
| Utilisation secondary care (short) | 0.08 (-0.03 to 0.19) **^(13)^** | 0.03 (-0.10 to 0.15) **^(5)^** |
| Utilisation secondary care (long) | 0.02 (-0.06 to 0.09) **^(7)^** | -0.10 (-0.22 to 0.0) **^(6)^** |
|  | **Low PHC score ^(18)^** | **Int/high PHC score ^(13)^** |
| Mortality (short) | n/a | n/a |
| Mortality (long) | 0.04 (-0.02 to 0.11) **^(8)^** | -0.10 (-0.27 to 0.08) **^(3)^** |
| Self-rated health (short) | 0.12 (-0.05 to 0.29) **^(4)^** | 0.03 (-0.08 to 0.13) **^(6)^** |
| Utilisation primary care (short) | -0.04 (-0.19 to 0.11) **^(8)^** | -0.00 (-0.20 to 0.20) **^(5)^** |
| Utilisation secondary care (short) | 0.03 (-0.05 to 0.10) **^(11)^** | 0.08 (-0.10 to 0.26) **^(7)^** |
| Utilisation secondary care (long) | -0.05 (-0.15 to 0.05) **^(8)^** | -0.02 (-0.12 to 0.07) **^(5)^** |
|  | **Clinical Judgement ^(4)^** | **Risk modelling ^(27)^** |
| Mortality (short) | n/a | n/a |
| Mortality (long) | -0.02 (-0.30 to 0.26) **^(2)^** | 0.01 (-0.06 to 0.08) **^(9)^** |
| Self-rated health (short) | n/a | n/a |
| Utilisation primary care (short) | n/a | n/a |
| Utilisation secondary care (short) | -0.06 (-0.18 to 0.06) **^(3)^** | 0.09 (-0.00 to 0.18) **^(15)^** |
| Utilisation secondary care (long) | -0.01 (-0.15 to 0.14) **^(3)^** | -0.04 (-0.13 to 0.04) **^(10)^** |
|  | **RCT ^(23)^** | **Non-RCT ^(8)^** |
| Mortality (short) | n/a | n/a |
| Mortality (long) | 0.01 (-0.06 to 0.08) **^(8)^** | -0.00 (-0.18 to 0.17) **^(3)^** |
| Self-rated health (short) | n/a | n/a |
| Utilisation primary care (short) | n/a | n/a |
| Utilisation secondary care (short) | 0.05 (-0.04 to 0.13) **^(14)^** | 0.17 (-0.11 to 0.45) **^(4)^** |
| Utilisation secondary care (long) | -0.02 (-0.10 to 0.07) **^(9)^** | -0.08 (-0.19 to 0.02) **^(4)^** |
|  | **Social worker ^(10)^** | **No social worker ^(21)^** |
| Mortality (short) | n/a | n/a |
| Mortality (long) | 0.03 (-0.07 to 0.13) **^(2)^** | -0.00 (-0.09 to 0.08) **^(9)^** |
| Self-rated health (short) | 0.14 (-0.02 to 0.30) **^(4)^** | 0.02 (-0.07 to 0.10) **^(6)^** |
| Utilisation primary care (short) | -0.02 (-0.23 to 0.18) **^(8)^** | 0.01 (-0.09 to 0.11) **^(5)^** |
| Utilisation secondary care (short) | 0.11 (-0.01 to 0.22) **^(8)^** | 0.03 (-0.07 to 0.13) **^(10)^** |
| Utilisation secondary care (long) | -0.12 (-0.43 to 0.19) **^(2)^** | -0.02 (-0.10 to 0.05) **^(11)^** |

No significant difference between subgroups (p<0.05)

* = significant in-subgroup effect (p<0.05)

*Note*: Positive effect size favours case management for all measures
